# Vascular Stem/Progenitor Cell Migration Induced by Smooth Muscle Cell‐Derived Chemokine (C‐C Motif) Ligand 2 and Chemokine (C‐X‐C motif) Ligand 1 Contributes to Neointima Formation

**DOI:** 10.1002/stem.2410

**Published:** 2016-06-28

**Authors:** Baoqi Yu, Mei Mei Wong, Claire M. F. Potter, Russell M. L. Simpson, Eirini Karamariti, Zhongyi Zhang, Lingfang Zeng, Derek Warren, Yanhua Hu, Wen Wang, Qingbo Xu

**Affiliations:** ^1^ Cardiovascular DivisionKing's College London BHF CentreLondonUnited Kingdom; ^2^ Institute of Bioengineering, School of Engineering and Materials Science, Queen Mary University of London, LondonUnited Kingdom; ^3^ The Key Laboratory of Cardiovascular Remodelling and Function Research, Chinese Ministry of Education and Chinese Ministry of Health, Qilu Hospital, Shandong UniversityJinanChina

**Keywords:** Vascular stem/progenitor cells, Smooth muscle cells, CCL2, CXCL1, Migration

## Abstract

Recent studies have shown that Sca‐1^+^ (stem cell antigen‐1) stem/progenitor cells within blood vessel walls may contribute to neointima formation, but the mechanism behind their recruitment has not been explored. In this work Sca‐1^+^ progenitor cells were cultivated from mouse vein graft tissue and found to exhibit increased migration when cocultured with smooth muscle cells (SMCs) or when treated with SMC‐derived conditioned medium. This migration was associated with elevated levels of chemokines, CCL2 (chemokine (C‐C motif) ligand 2) and CXCL1 (chemokine (C‐X‐C motif) ligand 1), and their corresponding receptors on Sca‐1^+^ progenitors, CCR2 (chemokine (C‐C motif) receptor 2) and CXCR2 (chemokine (C‐X‐C motif) receptor 2), which were also upregulated following SMC conditioned medium treatment. Knockdown of either receptor in Sca‐1^+^ progenitors significantly inhibited cell migration. The GTPases Cdc42 and Rac1 were activated by both CCL2 and CXCL1 stimulation and p38 phosphorylation was increased. However, only Rac1 inhibition significantly reduced migration and p38 phosphorylation. After Sca‐1^+^ progenitors labeled with green fluorescent protein (GFP) were applied to the adventitial side of wire‐injured mouse femoral arteries, a large proportion of GFP‐Sca‐1^+^‐cells were observed in neointimal lesions, and a marked increase in neointimal lesion formation was seen 1 week post‐operation. Interestingly, Sca‐1^+^ progenitor migration from the adventitia to the neointima was abrogated and neointima formation diminished in a wire injury model using CCL2^−/−^ mice. These findings suggest vascular stem/progenitor cell migration from the adventitia to the neointima can be induced by SMC release of chemokines which act via CCR2/Rac1/p38 and CXCR2/Rac1/p38 signaling pathways. Stem Cells *2016;34:2368–2380*

**Significance Statement**

In the present article, we provide the first evidence that vascular smooth muscle cell produced chemokine CCL2 and CXCL1 play a key role in mediating resident stem cell migration from the adventitia to intima, where they are composed of neointimal lesions in mouse model. This can be reverted by the chemokine gene CCL2 knockout. We feel that our findings provide a new insight into the mechanisms of vascular stem cell migration with a therapeutic potential for restenosis, which would be interesting for the readers.

## Introduction

Smooth muscle cells (SMCs) are well established as a key cell type that can contribute to the pathology of vascular diseases such as atherosclerosis and post‐angioplasty restenosis 1, 2, 3. Increasing evidence suggests that during vascular injury, SMCs in the media layer of the vessel undergo dedifferentiation from a quiescent/contractile phenotype to an active/synthetic phenotype and contribute to neointima formation through increased cell proliferation and migration 4, 5. During this process, SMCs secrete a variety of chemokines such as chemokine (C‐C motif) ligand 2 (CCL2) and (C‐X‐C motif) ligand 1 (CXCL1) 6. These chemokines are largely responsible for recruitment of inflammatory cells 7, 8. For example, after vascular injury, CCL2 is released by SMCs in a hypercholesterolemia mouse model and simultaneously chemokine (C‐C motif) receptor 2 (CCR2) is upregulated on monocytes, which leads to over‐recruitment of leukocytes to lesion sites 9, 10. In ApoE^−/−^mice CXCL1 presented by the lesion‐prone endothelium triggers monocyte recruitment in a process dependent on α_4_β_1_ integrin 11. In the late stage of atherosclerotic lesion development, CXCL1 is also important for macrophage accumulation via leukocyte expression of chemokine (C‐C motif) receptor 2 (CXCR2) 12. Additionally, neutralization of CXCL1 reduces endothelial chemotaxis in vitro and delays endothelial recovery after arterial injury in vivo in a CXCR2‐dependent manner 13. However, little is known about the roles of CCL2 and CXCL1 secreted by SMCs in recruitment of other cell types, for example, vascular resident stem/progenitor cells, into the intima in response to injury.

Recently, studies from several laboratories have identified the presence of a range of multipotent and lineage‐restricted stem/progenitor cells in the adventitia of the vessel wall. These cells possess high potential to differentiate into many cell lineages, including endothelial and SMCs, adipocytes and osteoblasts 14, 15, 16. Progenitor cells residing in the adventitia are positive for the stem/progenitor markers stem cell antigen‐1 (Sca‐1) and c‐kit 17. In our recent studies, we have shown that Sca‐1^+^ cells can migrate toward sirolimus, a drug used to coat stents 18, and differentiate into SMCs in vitro and in an ex vivo model of decellularized vessels 17, 19. However, it is unknown whether chemokines released from SMCs in response to injury play a role in attracting vascular stem cells. The aims of this study were to investigate whether local SMC‐released chemokines can induce adventitial‐derived resident progenitor cell migration and to elucidate the mechanism behind progenitor cell promotion of neointima formation after vessel injury. In the present report, we demonstrate that SMCs can induce Sca‐1^+^ progenitor cell migration upon the release of CXCL1 and CCL2, which in turn activate the Rac1/p38 MAPK signaling pathways via the receptors CXCR2 and CCR2 respectively. Importantly, we demonstrate that CCL2‐deficent mice display reduced Sca‐1^+^ cell migration and neointima formation in response to vessel injury.

## Materials and Methods

### Mouse Vascular Progenitor Cell Culture

The vena cava of a C57BL/6J mouse (Harlan, Blackthorn, Bicester, UK) was isografted to the carotid artery of an isogenic mouse or GFP C57BL/6J mouse (Jackson Laboratory, Bar Harbour, Maine, USA). After 2 weeks, graft tissues were harvested and explanted in gelatin‐coated flasks. Culture was in complete stem cell culture medium.

### Cell Sorting

Vascular progenitor cells (VPCs) were sorted with anti‐Sca‐1 immunomagnetic microbeads (Miltenyi Biotec, GmbH, Bergisch Gladbach, Germany) and then selected using a magnetic cell separator (Miltenyi Biotec, GmbH, Bergisch Gladbach, Germany). Sca‐1^+^ VPC populations were expanded for up to 5 population doublings.

### Statistical Analysis

Data for this study are presented as the mean ± standard error of the mean (SEM.) of at least three separate experiments. Analysis was performed using Graphpad Prism V.6 (GraphPad Software, San Diego, CA) using analysis of variance (one way ANOVA) followed by Dunnett's multiple comparison tests. Significance was considered when *p* < .05.

Detailed Methods are included in the Supporting Information Online Data.

## Results

### Characterization of Vascular Stem/Progenitor Cells

Previously published works by our group identified a population of progenitor cells within pathological vessels from a mouse vein graft model 19, 20. As in this previous work, 2 weeks after implantation of vein grafts, a large heterogeneous population of cells was found to migrate from the adventitia of vessels and contributed to neointima formation 17. Cells were isolated from the adventitia and used to derive clones of single cells. Immunofluorescent staining showed that cells from this cloned population express progenitor cell marker Sca‐1 (Supporting Information Fig. 1A, 1B), but express very few SMC or endothelial cell markers (Supporting Information Fig. 1C, 1D). In order to purify a larger pool of Sca‐1^+^ progenitor cells from the initially heterogeneous adventitial population anti‐Sca‐1 immunomagnetic microbeads were used. Approximately 15% of total cells collected using this method was identified as Sca‐1^+^ progenitor cells after sorting (Data not shown). Sca‐1^+^ cells derived from clones and a mixed population of Sca‐1^+^ progenitor cells derived by magnetic sorting were further characterized by a qPCR array showing gene expression of chemokines and chemokine receptors, further details are described in the following results. Our hypothesis is that Sca‐1^+^ progenitors may contribute to the pathology of atherosclerosis, potentially via their expression of chemokines and receptors.

### SMC‐Conditioned Medium Induced VPC Migration

**Figure 1:**
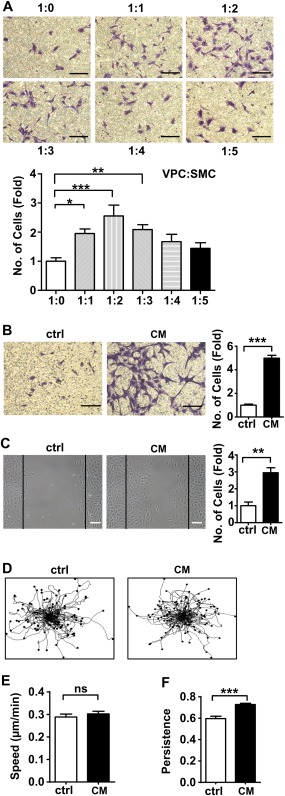
SMC conditioned medium can induce VPCs migration. **(A)**: Chemotaxis of vascular progenitor cells (5 × 10^4^ cells/well) across 8.0 μm transwells toward an increasing number of SMCs after 18 hours was documented after 0.1% crystal violet staining. Serum free culture medium was used as control for all migration experiments, *n* = 5. Scale bars, 100µm. **(B, C)**: Migration of vascular progenitor cells in response to SMC conditioned medium. Chemotaxis index was defined by average of 9 fields of view and was presented as fold increase compared to the control, *n* = 5. Scale bars, 100 µm. **(D–F)**: Vascular progenitor cells were incubated with or without SMC conditioned medium for 20 hours. Time lapse microscopy was performed to observe single cell movement. Trajectory plots of migrating VPC are shown in (D), 100 tracked single cells in each group are displayed. Quantification analysis of the speed (E) and persistence (F) of VPC were performed in 3 independent experiments. All graphs are shown as mean ± SEM. *p < .05, **p < .01, ***p < .001. ns, *p* > .05. Abbreviations: ctrl, control, serum free medium; CM, SMC conditioned medium; SMC, smooth muscle cell; VPCs, vascular progenitor cells.

To investigate whether SMCs influence the migratory capacity of VPCs, a transwell migration assay was performed. Increasing numbers of SMCs were seeded on the base of the lower chamber while fixed numbers of VPCs were seeded on the 8.0 µm pore membrane of the upper chamber. Using an increasing ratio of VPCs to SMCs (from 1:0 to 1:5), and allowing an overnight incubation, we found that the migration of VPCs increased as the number of SMCs increased and this reached its peak when the VPC:SMC ratio was 1:2 (Fig. 1A). Next, SMC‐conditioned medium was collected from SMC cultured overnight in serum free medium and used for migration assays. Addition of SMC‐conditioned medium consistently stimulated VPC migration in both transwell and wound healing assays, when compared to serum free medium. (Fig. 1B, 1C).

Time lapse microscopy was used to track the speed and the persistence of single progenitor cells during their migration when treated with SMC conditioned medium in an overnight incubation. These results showed that SMC‐conditioned medium augmented directional persistence of VPC migration, while migrational velocity remained unaltered between the treatments (Fig. 1D–1F and Supporting Information online Video 1 (control), Supporting Information online Video 2 (SMC conditioned medium)). Use of a BrdU cell proliferation assay confirmed that this effect was not due to proliferation (Supporting Information Fig. 2A). qPCR analysis revealed that the mRNA levels of SMC markers (calponin and α‐SMA) and endothelial marker (CD31) in VPCs were not significantly changed during the experiment (Supporting Information Fig. 2B–2D). VPCs also maintained progenitor characteristics, as the progenitor marker Sca‐1 was not significantly changed when compared to the control (Supporting Information Fig. 2E). Additionally, immunofluorescence staining for paxillin, vinculin and phosphorylated FAK showed that relocation of cytoskeleton‐related proteins was increased (Supporting Information Fig. 3A). Taken together, the above results support the notion that both coculture with SMC and SMC‐derived conditioned medium can induce VPC migration.

### SMC‐Released CCL2 and CXCL1 Induced VPC Migration

**Figure 2:**
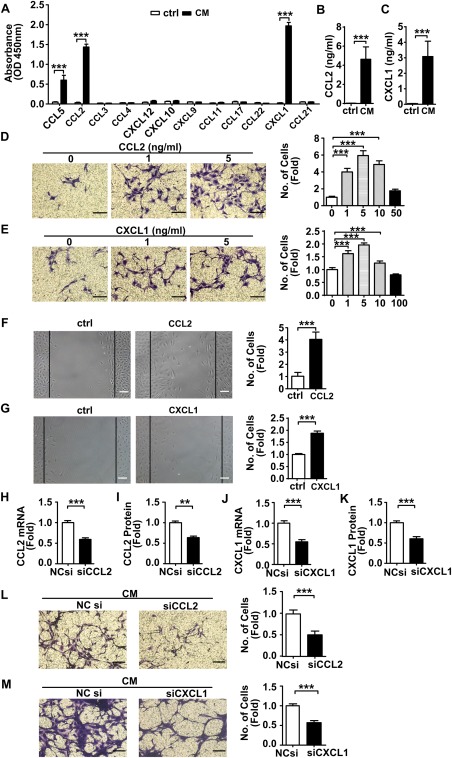
CCL2 and CXCL1 released from SMC conditioned medium induced VPCs migration. **(A)**: The identification of chemokines in SMC conditioned medium using a Chemokine Multiarray ELISA kit. The graph indicates the absorbance of each chemokine at 450 nm. n = 3. **(B, C)**: The concentrations of CCL2 and CXCL1 in SMC conditioned medium were quantified by the murine CCL2 or CXCL1 Quantikine ELISA kit. *n =* 3. **(D, E)**: Changes in vascular progenitor cells migration in response to a gradient of CCL2 or CXCL1 in serum free culture medium were evaluated using a transwell assay. *n* = 5. Scale bars, 50 µm. **(F, G)**: Wound healing assay was performed on vascular progenitor cells treated with CCL2 (5 ng/ml), CXCL1 (5 ng/ml) or vehicle control. Graphs are shown as fold increase relative to controls. Scale bars, 100 µm. SMCs were transfected either with control noncoding small interfering RNA (siRNA), CCL2 siRNA (300 nM) or CXCL1 siRNA (100 nM) to knockdown corresponding mRNA. The real‐time quantitative PCR and Quantikine ELISA kit showed the folds decrease in mRNA **(H, J)** and protein **(I, K)** levels in CCL2 or CXCL1 siRNA transfected SMCs and their conditioned medium respectively. *n =* 3. **(L, M)**: Transwell assay was performed on vascular progenitor cells migrating toward SMC (transfected either with noncoding siRNA, CCL2 siRNA or CXCL1 siRNA) conditioned medium. *n =* 5. Scale bars, 50µm. All graphs are shown as mean ± SEM. **p < .01, ***p < .001. Abbreviations: ctrl, control, serum free medium; NCsi, noncoding siRNA; siCCL2, CCL2 siRNA; siCXCL1, CXCL1 siRNA; CM, SMC conditioned medium; SMC, smooth muscle cell.

To further investigate which mediators released by SMCs were responsible for the conditioned medium induced VPC migration, we performed a chemokine multi‐ELISA array. This revealed that CXCL1 and CCL2 levels were markedly increased in SMC‐conditioned medium when compared to serum free medium (Fig. 2A). Murine CCL2 or CXCL1 Quantikine kits established that the amounts of CCL2 and CXCL1 in the conditioned medium were 4.63 ± 1.29 ng/ml and CXCL1 3.08 ± 0.99 ng/ml, respectively, (Fig. 2B, 2C). While the level of CCL5 was also upregulated in comparison to the control, subsequent transwell migration assays found that it did not affect VPC migration (Supporting Information Fig. 4A, 4B). To confirm the roles of CCL2 and CXCL1 in mediating VPC migration, we performed transwell and wound healing assays using exogenous mouse recombinant CCL2 and CXCL1 proteins, respectively. VPC migration was enhanced by CCL2 and CXCL1 treatment and peaked at 5 ng/ml (a sixfold increase in CCL2 and a twofold increase in CXCL1 treated cells) (Fig. 2D, 2E). These were similar concentrations of CCL2 and CXCL1 to those measured in SMC‐derived conditioned medium. That a 5 ng/ml CCL2 or CXCL1 treatment enhanced VPC migration was confirmed by wound healing assay (Fig. 2F, 2G). Silencing of CCL2 or CXCL1 in the SMCs using siRNAs resulted in significant down‐regulation of CCL2 and CXCL1 at both the mRNA and protein levels (Fig. 2H–2K). Importantly, CCL2 or CXCL1 depleted SMCs failed to stimulate VPC migration in transwell assays (Fig. 2L, 2M). To further investigate the relationship between CCL2 and CXCL1 in SMC conditioned medium induced VPC migration, CCL2 and/or CXCL1 concentrations were depleted using their corresponding neutralizing antibodies. The antibodies were found to be effective and selective as they significantly reduced the availability of their target chemokine while they had no effect on the other chemokine studied. Depletion of either CCL2 or CXCL1 did not result in a compensatory release of the other chemokine and there appeared to be no interactions between the two antibodies (Supporting Information Fig. 5A, 5B). Having confirmed the effectiveness of the neutralizing antibodies they were used to study the role of CCL2 and CXCL1 in VPC migration. Depletion of either CCL2 or CXCL1 in SMC conditioned medium inhibited migration of VPCs by nearly 50%. However, simultaneous depletion of CCL2 and CXCL1 in SMC conditioned medium did not further reduce this migration level (Supporting Information Fig. 5C, 5D). Finally, incubation with various concentrations of CCL2 or CXCL1 did not stimulate VPCs autocrine production of the other chemokine (Supporting Information Fig. 5E, 5F). Taken together, these results suggest that although CCL2 and CXCL1 derived from SMCs play an important role in the induction of VPC migration, their interaction is neither cumulative nor redundant but both are required for migration to be induced.

### VPC Migration is Mediated by CCR2 and CXCR2

**Figure 3:**
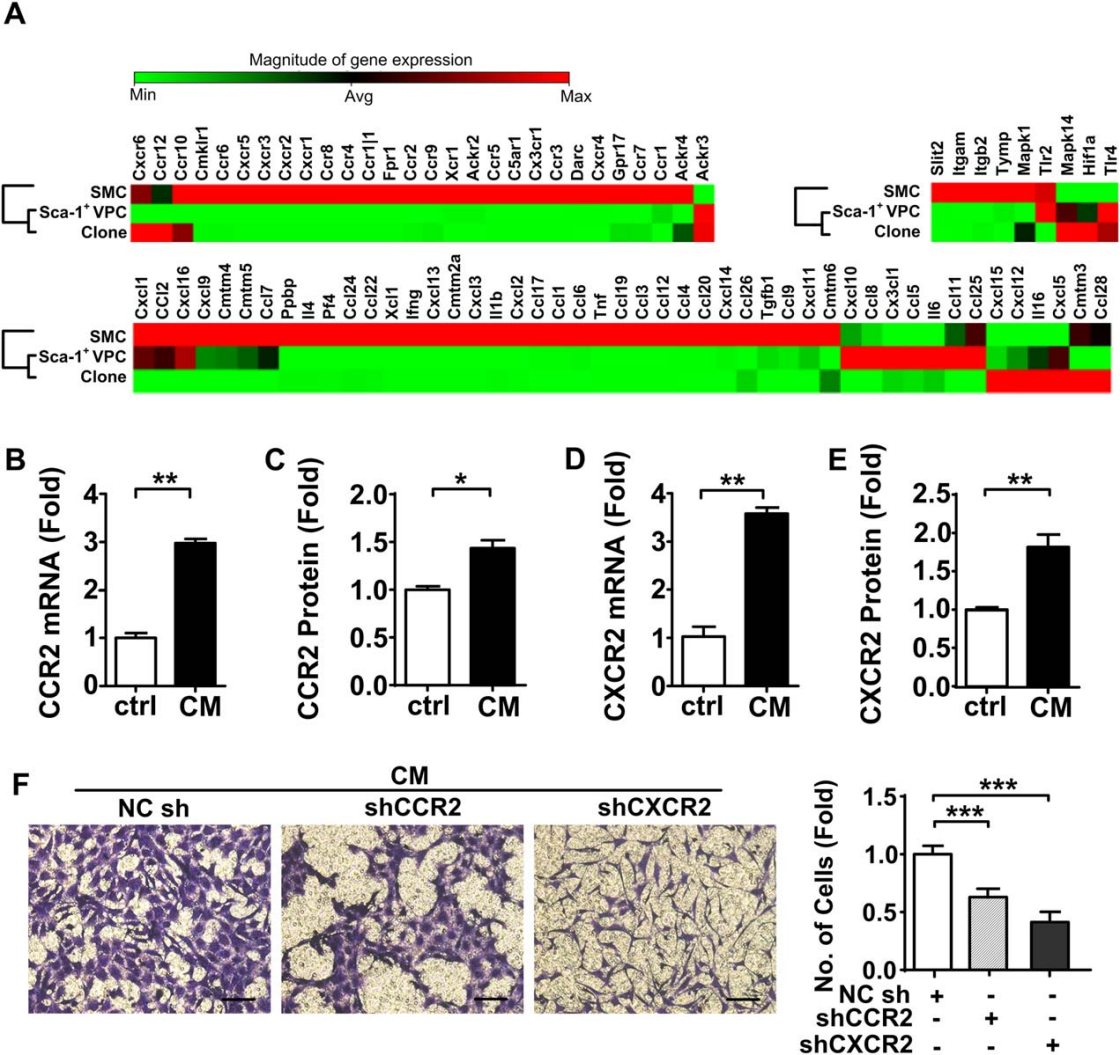
SMC conditioned medium induced VPC migration by up‐regulating expression of CCR2 and CXCR2. **(A)**: Cluster grams show the expression of 84 chemokine receptors, chemokine and other chemotactic cytokines on the untreated smooth muscle cells, clones and Sca‐1^+^ VPCs individually. A row in the cluster represents a cell line, and relative level of expression for a gene is arranged in column. The magnitude of gene expression increases from green to red. The dendrogram of the different cell lines clustering is displayed aside and describes the degree of relatedness among different cell lines. Quantification was performed with three different batches of each cell line. The vascular progenitor cells were treated with or without SMC conditioned medium for 18 hours, untreated cells served as controls. *n* = 3. The mRNA **(B, D)** and cell‐surface protein **(C, E)** expression of CCR2 or CXCR2 was confirmed using real‐time quantitative PCR and flow cytometry. *n* = 3. **(F)**: Vascular progenitor cells were infected with lentiviral short hairpin RNA (shRNA) (noncoding shRNA, CCR2 shRNA or CXCR2 shRNA) for ablation of CCR2 or CXCR2 before migration toward SMC conditioned medium. The noncoding shRNA served as the control. *n* = 3. Scale bars, 100 µm. All graphs are shown as mean ± SEM. *p < .05, **p < .01, ***p < .001. Abbreviations: CM, SMC conditioned medium; ctrl, control, serum free medium; NCsh, noncoding shRNA; shCCR2, CCR2 shRNA; shCXCR2, CXCR2 shRNA; SMC, smooth muscle cell; VPCs, vascular progenitor cells.

Each chemokine needs to interact with its corresponding receptor in order to exert an effect. We investigated the chemokine receptor profile of VPCs by performing a qPCR‐array of a mixed population of Sca‐1^+^ progenitor cells derived by magnetic sorting and Sca‐1^+^ cells derived from a clone. We demonstrated that VPCs possess a variety of chemokine receptors (Fig. 3A, Supporting Information Table 2) and importantly, CCR2 and CXCR2, the corresponding receptors of CCL2 and CXCL1. Both mRNA and protein level expression of these receptors was significantly upregulated after treatment with SMC conditioned medium (Fig. 3B–3E). To silence CCR2 and CXCR2 in VPCs at the gene level, VPCs were infected with CCR2 or CXCR2 lentiviral shRNA while a null shRNA was also introduced into VPCs as a control. The cells were selected for 4‐5 days using neomycin and maintained in complete culture medium. Subsequent transwell migration experiments with CCR2 or CXCR2 silencing resulted in a significant decrease in VPC migration (Fig. 3F). In addition, pretreatment with antagonists of CCR2 or CXCR2 led to downregulation of VPC migration in response to chemokines (Supporting Information Fig. 6A, 6B). These findings suggest that CCL2 and CXCL1 released from SMCs induce VPC migration via interaction with their corresponding receptors CCR2 and CXCR2.

### CCL2 and CXCL1 Induced VPCs Migration Through the Rac1‐p38 Pathways

**Figure 4:**
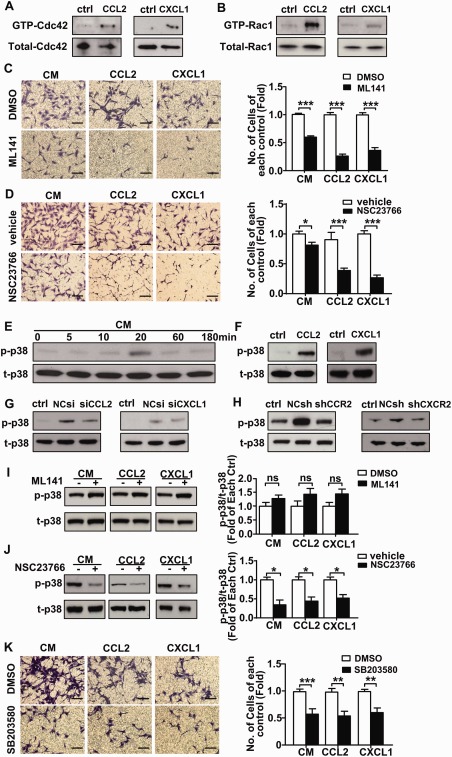
Smooth muscle cell (SMC) induces vascular progenitor cells (VPCs) migration via activated GTPase Cdc42 and Rac1 and p38 signaling pathway. **(A, B)**: Pull down assays were performed on VPCs treated with either CCL2 or CXCL1 for 5 minutes. The transwell assay was performed on VPCs that were pretreated with either vehicle, DMSO or ML141 (20 µM) **(C)**, NSC23766 (50 µM) **(D)**, SB203580 (10µM) **(K)** for 1 hour before migration toward either SMC conditioned medium or recombinant CCL2, CXCL1. Scale bars, 100µm. The quantification of transwell assay shows as the fold of changes compared with cell migrated toward each treatment pre‐treated with vehicle or DMSO. Western blotting was performed on SMC conditioned medium **(E)**, CCL2 or CXCL1 **(F)**, SMCs (transfected with CCL2 siRNA or CXCL1 siRNA) conditioned medium **(G)**‐treated VPCs for the detection of p‐p38 and t‐p38. **(H)**: Cell lysates from CCR2 shRNA or CXCR2 shRNA transfected VPCs cultured in the SMC conditioned medium were harvested for the detection of the p‐p38 and t‐p38. Untreated cells served as the controls. **(I, J)**: After pre‐treated with ML141, NSC23766 for 1 hour, VPC were stimulated by SMC conditioned medium or CCL2, CXCL1 before cell lysates were harvested for detection of the p‐p38 and t‐p38. All the blots shown are representative of three separate experiments. All graphs are shown as mean ± SEM. *n =* 3. *p < .05, **p < .01, ***p < .001. Abbreviations: CM, SMC conditioned medium; ctrl, control, serum free medium; DMSO, dimethyl sulfoxide; NCsh, noncoding shRNA; NCsi, noncoding siRNA; p‐p38, phosphorylated p38; shCCR2, CCR2 shRNA; shCXCR2, CXCR2 shRNA; siCCL2, CCL2 siRNA; siCXCL1, CXCL1 siRNA; t‐p38, total p38; vehicle, sterile distilled water.

The Rho GTPase family members Rac1 and Cdc42 have been implicated as important regulators of cell migration 21. To investigate whether Cdc42 and Rac1 are involved in VPC migration, we measured the level of GTP‐Cdc42 and GTP‐Rac1 using pull down assays. Stimulation of VPCs with mouse recombinant CCL2 or CXCL1 resulted in both Cdc42 and Rac1 activation (Fig. 4A, 4B). Treatment with either ML141 (an inhibitor of Cdc42) or NSC23766 (an inhibitor of Rac1) resulted in a significant reduction in VPC migration induced by CCL2 and CXCL1 as compared to control (Fig. 4C, 4D).p38 MAPK has been identified as the down‐stream signal transducer of active forms of Cdc42 and Rac1 and the p38 signaling pathway can induce rearrangements of the cytoskeleton that regulate cell migration 22, 23. A time course using Western blotting analysis showed p38 phosphorylation occurred in VPCs after treatment with SMC conditioned medium for 20 minutes (Fig. 4E). p38 phosphorylation was upregulated in response to mouse recombinant CCL2 and CXCL1 treatment (Fig. 4F), and suppressed in response to conditioned medium derived from CCL2 siRNA or CXCL1 siRNA depleted SMCs (Fig. 4G). p38 phosphorylation was also suppressed in VPCs where CCR2 or CXCR2 had been knocked down using the corresponding shRNA (Fig. 4H). Pretreatment of VPCs with CCR2 or CXCR2 antagonists consistently reduced the p38 phosphorylation (Supporting Information Fig. 6C, 6D). Importantly, p38 phosphorylation was significantly reduced in VPCs pretreated with NSC23766 but not ML141 (Fig. 4I, 4J). Finally, migration of VPCs was reduced in the presence of SB203580 (an inhibitor of the p38‐MAPK signaling pathway) (Fig. 4K). These indicate Rac1 regulates CCL2 and CXCL1 induced VPC migration via the p38 signaling pathway. RhoA, as another important small GTPase has also been reported to regulate the cell migration process 21, however, pretreatment of VPCs with C3 transferase (an inhibitor of RhoA), did not alter VPC migration or p38 phosphorylation (Supporting Information Fig. 7). We, therefore, conclude that CCL2 and CXCL1 induce VPC migration by activating GTPase Rac1 and p38 signaling pathways.

### Lack of CCL2 Inhibits Sca‐1^+^ Cell Migration and Neointima Formation

**Figure 5:**
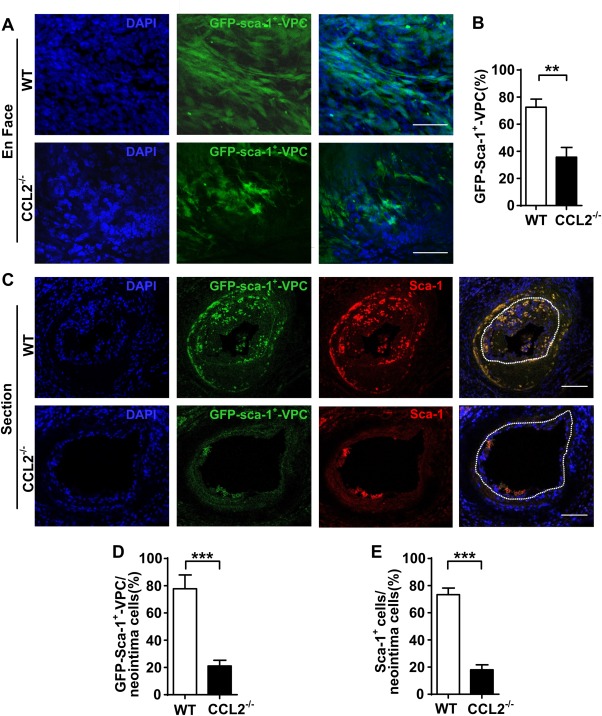
Lack of CCL2 inhibits Sca‐1^+^ cell migration in vivo. **(A)**: Using a mouse femoral artery wire injury model, GFP‐Sca‐1^+^‐VPC (1 x 10^6^) were seeded in the adventitia of each injured vessel. *En face* staining shows the cells were migrated to the intima side of the vessels 72hrs post injury of WT and CCL2^−/−^ mice. Scale bars, 25 µm. **(B)**: The percentage of GFP‐Sca‐1^+^‐VPC within respective DAPI^+^ populations in each view was quantified. **(C)**: The femoral arteries sections from WT and CCL2^−/−^ mice 2 weeks post injury were prepared for immunofluorescent Sca‐1 staining. Scale bars, 50µm. **(D, E)**: The graphs show the percentage of GFP‐Sca‐1^+^‐VPC or Sca‐1^+^ cells within the DAPI^+^ cells in the neointima (white dotted line indicates internal elastin, the neointima area was surrounded by the line). Representative images and graphs shown as mean ± SEM of *n =* 8 mice/group. **p < .01, ***p < .001. Abbreviations: GFP‐Sca‐1^+^‐VPC, GFP‐Sca‐1^+^ vascular progenitor cells; Sca‐1^+^, stem cell antigen‐1; WT, wild type.

SMCs can be described as activated and switched into the synthetic phenotype when they are cultured in vitro 24 and under these conditions they constitutively release CCL2 and CXCL1. To study chemokine release in in vivo conditions, a mouse femoral artery wire injury model was used to assess whether native SMCs only produce chemokines which attract VPC migration after cell injury. Coimmunofluorescence staining of α‐SMA and CCL2 or CXCL1 showed that in intact vessels, SMCs are quiescent and do not produce CCL2 or CXCL1. However, once SMCs are injured they release increasing levels of CCL2 and CXCL1 (both at a short time point (6 hours) and a long time point (2 weeks) (Supporting Information Fig. 8). These chemokines could be potent and persistent attractants for VPC migration. To verify this, GFP‐Sca‐1^+^‐VPCs, additionally labeled using a Qtracker 655 Cell Labeling Kit, were seeded on the adventitia of vessels which were injured. *En face* confocal microscopy revealed that 72 hours after injury the number of migrated cells found on the intimal side of the vessel wall was significantly lower in CCL2^−/−^ mice when compared to WT mice (Fig. 5A, Supporting Information Fig. 10A). CCL2^−/−^ mice were identified by genotyping mice and measuring CCL2 levels in peripheral blood (Supporting Information Fig. 9A, 9B). Quantification based on either GFP‐Sca‐1^+^‐VPCs or Qtracker showed similar results (Fig. 5B, Supporting Information Fig. 10B). Sca‐1 immunofluorescence staining in sections of injured arteries 2 weeks postinjury, showed that GFP‐Sca‐1^+^‐VPCs remained Sca‐1 positive after 2 weeks in vivo but that fewer migrated into the intimal side to contribute to neointima formation in CCL2^−/−^ mice compared to the WT mice (Fig. 5C–5E). These results suggest a role for CCL2 in VPC migration from the adventitia to the intima where they may contribute to neointima formation.

**Figure 6:**
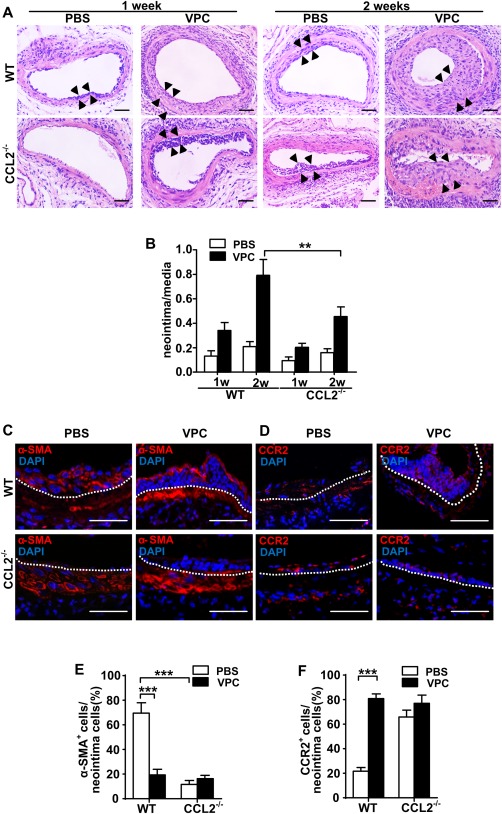
Lack of CCL2 reduces neointima formation and inhibits stem cell antigen‐1 cells migration and differentiation into smooth muscle cell (SMCs). **(A)**: Animals were euthanized at indicated time points after injury, and the femoral arteries were fixed in 4% phosphate‐buffered (pH = 7.2) formaldehyde, embedded in paraffin, sectioned in 5 µm, and stained with hematoxylin–eosin. Scale bars, 50 µm. **(B)**: The ratio of neointima (the area between arrows) to media was quantified as shown in the graph. **(C, D)**: Vessel sections were also prepared for immunofluorescent α‐SMA and CCR2 staining 2 weeks postwire injury (white dotted line indicates internal elastin, and the above is neointima area). Scale bars, 50 µm. **(E, F)**: Quantification of the percentage of positively stained cells within the neointima was shown in graphs as mean ± SEM of *n* = 8 mice/group. **p < .01, ***p < .001. Abbreviations: PBS, phosphate buffered saline; VPC, vascular progenitor cell; WT, wild type.

To further investigate the role of CCL2 in VPC neointima formation, wire‐injured femoral arteries were seeded with VPCs or PBS in the adventitia. HE stained sections showed neointima formation in the vessel at 1 or 2 weeks after wire injury (Fig. 6A, 6B). Quantification of the data indicated that neointimal lesions were significantly increased in vessels seeded with VPCs. In the 2 week postinjury group, the difference in ratio of neointima to media with VPCs or PBS was higher than that in the 1 week group. However, neointimal lesions in CCL2^−/−^ mice were decreased in vessels with either VPCs seeded in the adventitia or PBS. There is a significant reduction in neointimal lesions area of vessels with VPC seeding in CCL2^−/−^ mice when compared to the WT mice at 2 weeks.

To characterize cells contributing to neointima formation, immunofluorescence staining of α‐SMA and Sca‐1 was used, which showed that the cells in the neointima of PBS treated vessels were approximately 70% α‐SMA^+^ and 10% Sca‐1^+^ 2 weeks after injury (Supporting Information Fig. 11). Staining of α‐SMA and CCR2 in Figure 6 revealed that cell numbers in neointima of VPC seeded vessels were increased, although fewer were α‐SMA^+^. These results indicate that in the PBS treated vessels, most cells contributing to neointima formation are α‐SMA^+^. These cells are either from media SMC migration and proliferation or from progenitor cell differentiation. When Sca‐1^+^ VPC were seeded in the adventitia, the ratio of α‐SMA^+^ cells was significantly decreased which implies that VPCs possess high potential to migrate and proliferate. Over a short time most cells maintained their progenitor cell characteristics, although several had differentiated into SMCs (Fig. 6C, 6E). CCR2 was more highly expressed on the cells in the neointima of VPC‐seeded vessels (Fig. 6D, 6F). These data suggest that VPCs applied to the adventitia significantly increase neointima formation after vessel injury, largely through their migration although with a lesser effect due to their differentiation into SMCs. Furthermore, lack of CCL2 markedly inhibits the effect of VPCs on neointima formation.

### Role of CCL2 Released from Nonbone Marrow Tissue

**Figure 7:**
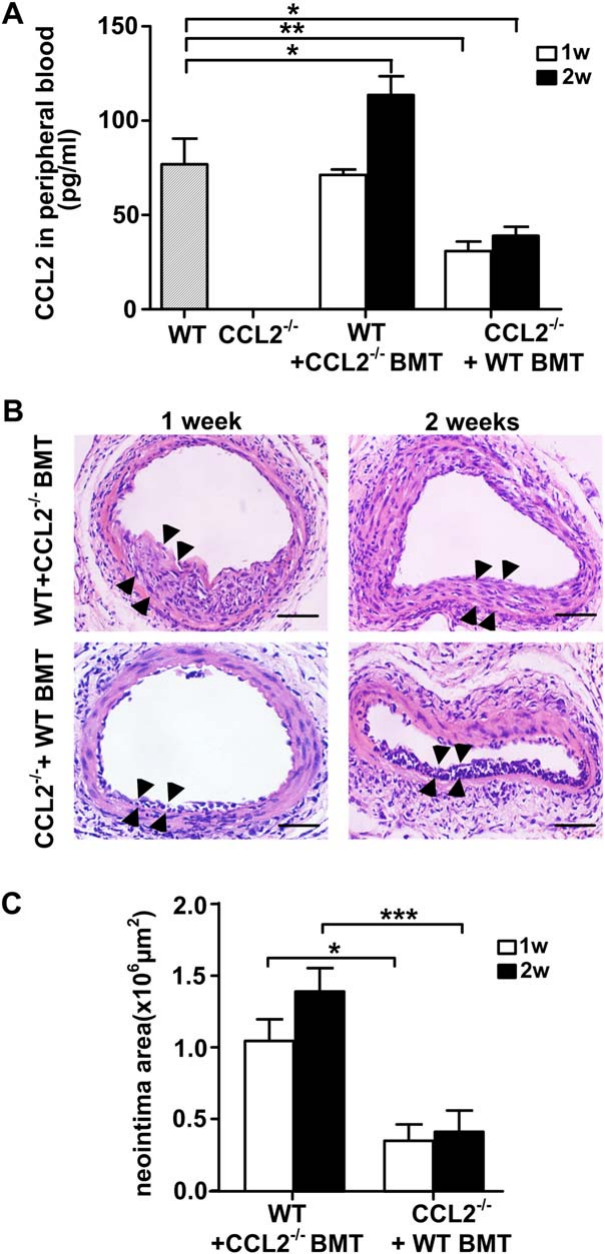
CCL2 released from peripheral tissue plays an important role in contribution of the neointima formation. **(A)** Using chimeric mice model, the CCL2 in peripheral blood of indicated mice was subjected to a CCL2 Quantikine ELISA kit. The vessels sections of indicated mice at various time points were stained with hematoxylin–eosin, and the neointima area (the area between arrows) was shown in the represented images **(B)** scale bars, 50µm., and quantified in the graphs **(C)** shown as mean ± SEM of *n* = 5 mice/group. *p < .05, **p < .01, ***p < .001. Abbreviations: WT + CCL2^−/−^ BMT, wild type mice of which bone marrow was transplanted from CCL2^−/−^mice; CCL2^−/−^+WT BMT, CCL2^−/−^ mice of which bone marrow was transplanted from wild type mice.

CCL2 is not only released from bone marrow cells but also from SMCs, endothelial cells, and other tissues 9, 25. To investigate which source of CCL2 plays the most important role in neointima formation due to VPC migration chimeric mouse models were created. Wild‐type bone marrow was transplanted into an irradiated CCL2^−/−^ mouse to form a CCL2 chimera. Conversely, a wild‐type chimeric mouse model was prepared by transplanting CCL2^−/−^ mouse bone marrow cells into an irradiated wild‐type mouse. The levels of CCL2 in serum of peripheral blood were measured using a murine CCL2 ELISA kit. In the wild‐type chimeric mouse model, the level of CCL2 in peripheral blood was increased despite transplantation with CCL2^−/−^ bone marrow when compared to a nontransplanted wild‐type mouse control, indicating that CCL2 is mostly released from nonbone marrow tissue. In contrast, the level of CCL2 was increased in CCL2^−/−^ mice with transplanted wild‐type bone marrow cells, but to a much lesser extent, indicating that only a small proportion of CCL2 is released from bone marrow derived cells (Fig. 7A). Data from HE stained sections showed that neointima lesion area correlated with the CCL2 level in blood (Fig. 7B, 7C). These results support the observations that CCL2 induced VPC migration contributes to neointima formation.

### CXCL1 Plays a Role in Sca‐1 Positive Cell Migration In Vivo

Previous studies have demonstrated that CXCL1 can induce endothelial cell migration and tube formation in vitro 26 and also acts as an angiogenic factor to promote tumor growth 27. To further investigate the role of CXCL1 in induction of VPC migration in vivo, VPCs were mixed with matrigel containing either PBS or mouse recombinant CXCL1 and injected subcutaneously. After 2 weeks, the number of cells that migrated into the matrigel was significantly increased in the CXCL1 group when compared to PBS controls (Supporting Information Fig. 12A). Immunofluorescence staining of Sca‐1, CD31 and α‐SMA showed that most of the migrated cells in the matrigel plug were Sca‐1 positive, whilst some were CD31 positive, and only very few were α‐SMA positive (Supporting Information Fig. 12B). In addition, CXCL1 or control siRNA within the pluronic gel was delivered to the adventitial side of wire injured vessels to assess the effect of local CXCL1 knockdown on Sca‐1^+^ VPC migration in vivo. Successful in vivo knockdown of CXCL1 mRNA level in injured femoral arteries was confirmed after 6 days using real time quantitative PCR (Supporting Information Fig. 13A). Furthermore, *En face* confocal microscopy revealed that 72 hours after seeding GFP‐Sca‐1^+^ VPC in the adventitia, the number of migrated cells found on the intimal side of the vessel wall was lower in CXCL1 siRNA treated vessels compared to the control siRNA (Supporting Information Fig. 13B). These results indicate the important role of CXCL1 in Sca‐1^+^ cells migration in vivo.

## Discussion

Restenosis is still the main complication that exacerbates the outcome of coronary artery disease after percutaneous coronary intervention 28, 29, 30. SMC proliferation and migration are suggested to be important factors in development of neointimal hyperplasia and restenosis 31. In the present study, we identify a new mechanism of smooth muscle accumulation in neointimal lesions after vascular injury, in which vascular stem/progenitor cells migrate from the adventitia to the intima. We demonstrate that proliferating SMCs can release several chemokines, including CXCL1 and CCL2, which have a role in attracting these vascular progenitors. Single cell tracking experiments indicate that cells are migrate directionally and efficiently but not randomly. Importantly, perivascular application of GFP‐Sca‐1^+^‐VPC to injured arteries significantly enhanced neointimal lesion formation via progenitor migration. This effect is diminished by CCL2 knockout. We provide the first evidence that the SMC‐produced chemokine CCL2 is crucial for vascular progenitor migration from the adventitia to the intima where these cells contribute to lesion formation.

After endothelial injury, an inflammatory response occurs in the vessel wall, and chemokines are released by both mononuclear cells and SMCs 32, 33. Using multiple chemokine ELISA we demonstrated that several chemokines were upregulated in cultured SMCs and amongst them, CXCL1, CCL2 and CCL5 were secreted at the highest levels. To further confirm the effects of these chemokines, we stimulated stem cells with exogenous mouse recombinant proteins and found that CCL2 and CXCL1 significantly induced vascular progenitor migration. Previous studies have reported that CCL5 mediates trafficking and homing of T cells, monocytes, basophils and eosinophils 34, 35, 36, 37. However, it does not appear to play such a role in induction of progenitor migration, indicating that vascular stem cells may selectively or specifically respond to certain chemokines.

Chemokine receptors are expressed on many different cell types, such as granulocytes, monocytes, mast cells, T cells, and endothelial cells 38, 39, but no prior study has shown the presence or absence of chemokine receptors on vascular progenitors. We used a qPCR array to profile the expression of chemokines, cytokines, and their receptors in different vessel wall cell lines. The results showed the presence of most cytokines and chemokine receptors in a mixed population of Sca‐1^+^ progenitor cells derived by magnetic sorting and one clone of Sca‐1^+^ progenitor cells and although SMCs display a higher expression level of different chemokine receptors. This distinct chemokine receptor profile marks a distinction between mature SMCs and progenitors. Expression of CCL2 and CXCL1 was much higher on SMCs compared to other cells, which was similar to our finding at the protein level of CCL2 and CXCL1 in SMCs as measured in the ELISA array. As many reports have demonstrated, CCL2/CCR2 and CXCL1/CXCR2 signal transduction mediates recruitment of neutrophils, monocytes or macrophages into inflammatory sites during progression of different kinds of disease 12, 40, 41, 42, 43. In our study, we found that both CCR2 and CXCR2 were upregulated on VPCs after treatment with SMC‐derived chemokines. The ability of VPCs to migrate after CCR2 and CXCR2 genes were permanently silenced by shRNA was significantly reduced. This indicates that SMC‐released CCL2 and CXCL1 activate their corresponding receptors on vascular stem/progenitor cells.

In order to elucidate the mechanism of cell migration, we studied the role of the Rho GTPase family. Rac1, Cdc42 and RhoA, as the main Rho GTPase family members, regulate the formation of lamellipodia, filipodia and focal adhesions, respectively 44. In the present study, we found both Cdc42 and Rac1 in VPCs were activated by CCL2 and CXCL1 and inhibition of either Cdc42 or Rac1 impaired VPC migration in response to CCL2 or CXCL1. Cytokines such as IL‐4 and TNF‐α can activate stress‐activated pathways leading to phosphorylation of p38 MAPK which is dependent on Rac1 and Cdc42 [45, 46]. In our study, p38 phosphorylation was upregulated in response to mouse recombinant CCL2/CXCL1, and p38 phosphorylation was suppressed by CCL2/CXCL1 knockdown or CCR2/CXCR2 inhibition. These results suggest that progenitor migration is induced through a p38 MAPK signaling pathway. Importantly, we also found that p38 phosphorylation was markedly downregulated by Rac1 inhibition but not inhibition of Cdc42 or RhoA, suggesting that progenitor migration is induced via a Rac1/p38 signaling pathway. Although the migration assay showed the participation of Cdc42 in SMC induced progenitor migration, it may through an as yet unidentified signaling pathway.

As the above results show that CCL2 and CXCL1 contribute to VPC migration equally, we wanted to further investigate possible interactions between the two chemokines. Depletion of each chemokine was found to partially inhibit VPCs migration, but treatment with each chemokine individually failed to stimulate VPCs to release the other. After loss of one chemokine, the induction of VPCs migration is not compensated by the other chemokine. Simultaneous depletion of both chemokines did not further inhibit VPC migration when compared to inhibition of a single chemokine. The effects of CCL2 and CXCL1 in the mediation of VPCs migration were found to be neither cumulative nor redundant. However, there may also be additional factors in SMC conditioned medium which play a role in inducing VPC migration, as migration was not completely abolished on depletion of both chemokines. We have shown that both chemokines induce VPC migration through the Rac1/p38 signaling pathway but act through their own receptors. Previous studies 47, 48 have shown that chemokine receptors can form coreceptors to mediate their effects, thus further studies need to be performed to determine whether CCR2 and CXCR2 form a coreceptor to induce VPC migration.

Reports using different animal models have shown that CCL2 plays an important role in neointimal hyperplasia 49, 50, 51, for example CCL2 enhances SMC migration, proliferation and invasion to remodel vessels 52, 53. VPCs have also previously been identified as playing a role in neointima formation 17, 19. In the present study, we demonstrated that migrating progenitors are an important cellular component of CCL2 mediated neointima formation. We found CCL2 not only enhanced VPC migration from the adventitia to the intima in a short time period but also accelerated neointima formation by inducing VPC migration. We also identified that the majority of cells contributing to neointima formation are not SMC marker positive but express a stem cell marker (Sca‐1), which indicates that although some VPCs participate in neointima formation and differentitate into SMCs, most of them retain progenitor cell characteristics as they migrate into the neointima.

The expression of CCR2 in neointimal cells suggests that the participtation of VPCs in neointima formation may be dependent on CCL2‐driven recruitment. Vande et al demonstrated the main source of CCL2 is bone marrow‐derived monocytes 54. In our study, through use of chimeric mouse models, we found that after bone marrow transplant, CCL2 can be detected in the peripheral blood of a CCL2^−/−^ chimeric mouse (bone marrow was from WT mouse), where it was originially undetectable. This confirms the findings of previous studies 54. However, in a WT chimeric mouse (bone marrow was from CCL2^−/−^ mouse), the levels of CCL2 were markedly upregulated and showed an even higher increase compared to the CCL2^−/−^ chimeric mouse. This indicates that CCL2 from nonbone marrow tissues (e.g., SMC) contributes more to the global CCL2 levels in the peripheral blood than bone marrow. Quantitative data from neointimal lesions further confirms that CCL2 from nonbone marrow tissues‐induces progenitor migration which contributes to neointima formation. Taken together, CCL2 released from nonbone marrow tissues (e.g., SMC) accelerates neointima formation through induction of progenitor migration from adventitia to neointima.

It was believed that neointimal formation in response to endothelial injury was mainly due to cell accumulation via recruiting inflammatory cells 55, 56 and SMCs 57, 58 from the media. In this study, we utilized an accelerated model of in vivo neointima formation by inducing endothelial injury. From the data generated in non‐VPC seeded groups, we found that during the short time period of 1‐2 weeks very few cells contributed to the spontaneous development of neointimal lesions. In contrast, an obvious neointima was found in VPC seeded groups, which suggested that the neointima formation is largely generated by VPCs migration in the early stage of the model. These findings have two implications. First, the cells accumulating in neointimal lesions may be derived from migration/proliferation of vascular stem cells, which can differentiate into SMC‐like cells, although mononuclear cells and medial SMCs contribute to the process. To obtain quantative data on how many cells in neointimal lesions are derived from progenitor cells, lineage tracing for progenitor cells in animal models would be essential. Secondly, a new treatment strategy for restenosis could be considered. Current treatments used in clinical are based on inhibition of cell proliferation 59, 60, for example, sirolimus. A recent report from our group suggests that sirolimus can enhance vascular stem/progenitor cell differentiation into SMCs, but inhibits endothelial differentiation 18. Our current findings that large numbers of stem/progenitor cells are recruited to the intima in response to CCL2 and CXCL1 provide a potential to direct cell differentiation into the endothelial lineage. In other words, a high number of stem cells exist during neointima formation, which could differentiate into endothelial cells if a new drug coated stent could harness their potential and directed them specifically.

## Summary

Taken together, these results may provide crucial answers as to why restenosis and delayed re‐endothelialization persist after angioplasty and stenting, which may be fundamental for developing new drugs that can improve the long‐term outcome of patients.

## Author Contributions

B.Y.: design, collection and assembly of data, data analysis and interpretation, manuscript writing; M.M.W.: design, data analysis and interpretation, manuscript modification; C.M.F.P., R.M.L.S., E.K., and D.W.: collection and assembly of data, manuscript modification; Z.Z.: collection and assembly of data; L.Z.: data interpretation, manuscript modification; Y.H.: collection and assembly of data; W.W.: conception and design, administrative support; Q.X.: conception and design, financial support, administrative support, data analysis and interpretation, manuscript modification, final approval of manuscript.

## Disclosure of Potential Conflict of Interest

The authors indicate no potential conflicts of interest.

## Supporting information

Additional Supporting Information may be found in the online version of this article

Supporting Information

Supporting Information Video 1.

Supporting Information Video 2.

Supporting Information Table 1.

Supporting Information Table 2.

Supporting Information Table 3.
