# Supplementary material for: Vascular Stem/Progenitor Cell Migration Induced by Smooth Muscle Cell‐Derived Chemokine (C‐C Motif) Ligand 2 and Chemokine (C‐X‐C motif) Ligand 1 Contributes to Neointima Formation
Source: Stem Cells. 2016 Jun 28;34(9):2368–80. doi: 10.1002/stem.2410 (PMC5026058; doi:10.1002/stem.2410)
Supplement: Supplementary file 1 — Supporting Information [file STEM-34-2368-s001.doc]

**SUPPLEMENATAL MATERIAL**

**Vascular Stem/Progenitor Cell Migration Induced by SMC-derived CCL2 and CXCL1 Contributes to Neointima Formation**

Running title: Vascular stem cell migration

**Baoqi Yu, Mei Mei Wong, Claire MF Potter, Russell ML Simpson, Eirini Karamariti, Zhongyi Zhang,** **Lingfang Zeng, Derek Warren, Yanhua Hu, Wen Wang and Qingbo Xu**

**SUPPLEMENTAL MATERIAL AND METHODS**

**Mice**

All procedures were performed according to protocols approved by the Institutional Committee for Use and Care of Laboratory Animals. The CCL2 deficient mice (B6.129S4-*Ccl2^tm1Rol^*/J) and GFP mice (C57BL/6-Tg(UBC-GFP)30Scha/J) on the C57BL/6J background were purchased from The Jackson Laboratory (Bar Harbour, Maine, USA). C57BL/6J mice (as WT mice) were purchased from Harlan, UK. Genotyping for CCL2^+/-^ and CCL2^-/-^ mice was performed using standard PCR according to a protocol provided by The Jackson Laboratory. The following primers were used: oIMR7415 (CCL2^-/-^ forward) 5’- GCC AGA GGC CAC TTG TGT AG-3’, oIMR9219 (CCL2^+/+^ forward) 5’- TGA CAG TCC CCA GAG TCA CA’ and oIMR9220 (common reverse) 5’- TCA TTG GGA TCA TCT TGC TG -3’.

**Mouse vascular progenitor cell culture**

Mouse vascular progenitor cells were derived from the outgrowth of adventitial tissues of vein grafts as previously described[1]. Briefly, the vena cava from a C57BL/6J mouse was isografted between two ends of the carotid artery of an isogenic mouse or GFP C57BL/6J mouse. After 2 weeks graft tissues were carefully harvested and cut into pieces and explanted on 0.04% gelatin (0.04% of 2% Solution Type B from Bovine Skin, Sigma)-coated flasks. Culture was in complete stem cell culture medium comprised of Dulbecco's Modified Eagle's Medium (ATCC, Rockville, Massachusetts, USA) supplemented with 10% ES Cell Qualified Fetal Bovine Serum (EmbryoMax, Millipore), 10 ng/ml leukemia inhibitory factor, 0.1 mM β-mercaptoethanol, 100U/ml penicillin/streptomycin and 2mM L-glutamine(Life Technologies). Graft tissues were incubated at 37℃，5% CO_2_ for 3 days. The cells that grew out from the graft tissue were dissociated from the flasks using 0.05% trypsin-EDTA (Life Technologies) and were passaged every other day at a 1 to 3 ratio.

**Isolation of clones and characterization**

Single cell clones were isolated by serial dilution of vascular progenitor cells, as described on the Corning Incorporated website[2]. Briefly, 96 wells (two 48-well plates) were labeled from rows A to H and columns 1 to 12. 200μl of culture medium was added to each of the wells except well A1. 400μl cell suspension was then added to A1, and 200μl was transferred from A1 to B1. These 1:2 dilutions were repeated down the column until well H1, discarding 200μl from H1. 200μl was then transferred from the wells in the first column (A1 to H1) to those in the second column (A2 to H2). These 1:2 dilutions were repeated across the whole 96 wells, discarding 200μl from each well in the last column. Plates were then incubated at 37℃， in a 5% CO_2_ incubator. On the next day, wells that contained only a single cell were marked and medium was changed every other day. 7 to 10 days later, the single colony could be sub-cultured successively into a single well of a 24-well plate, a 12-well plate, or a T25 flask. The number of clones that were successfully expanded was recorded. Clones were characterized using conventional PCR and immunofluorescent staining.

**Mouse vascular smooth muscle cell culture**

Mouse vascular smooth muscle cells were isolated from the aortas of C57BL/6J mice as described previously[3]. In brief, the murine aortas were removed from the aortic arch to the thoracic aorta and washed within DMEM. The intima and inner two thirds of the media were carefully dissected from the aortas and cut into pieces, then placed onto a gelatin-coated (0.04%) plastic flask. The cells were cultured in Dulbecco's Modified Eagle's Medium (Life Technologies, Inc.) supplemented with 10% fetal bovine serum (Gibico 10270) and 100U/ml penicillin/streptomycin. Cells were incubated at 37°C in a humidified atmosphere of 5% CO_2_. The medium was changed every 2 days. The purity of SMCs was routinely confirmed by immunostaining with α-SMA, SM-22 α and SM-MHCII antibodies. Smooth muscle cell conditional medium was collected from the supernatant of serum free medium, in which cells had been cultured overnight, by centrifuging 1500rpm, 15mins, at 4℃.

**Antibodies**

Primary antibodies were mouse anti-Cdc42, mouse anti-Rac1 (Millipore 17-441 | Rac1/Cdc42 Activation Assay Kit), rabbit anti- Phospho-p38 MAPK (Thr180/Tyr182) (Cell signaling 4511), rabbit anti-p38 MAPK (Cell signaling 9212), rat anti-Sca-1/ Ly6A/E (abcam 51317), mouse anti-Actin, α-Smooth Muscle-Cy3 (α-SMA) (Sigma C6198), rabbit anti-calponin (abcam ab46794), rabbit anti-CCR2 (abcam ab21667), rabbit anti-vinculin (abcam ab73412), rabbit anti-paxillin (abcam ab32084) and goat anti-PECAM-1(Santa Cruz sc1506), goat anti-VE-cadherin (Santa Cruz sc-6458), mouse anti-CCL2 (R&D AF-479-SP), mouse anti-CXCL1 (R&D AF-453-SP). Secondary antibodies for immunostaining were anti-mouse Alexa Fluor 546, anti-rabbit Alexa Fluor 546 and anti-rat Alexa546 and were purchased from Invitrogen. Cells were also counterstained with Alexa Fluor® 488 Phalloidin (Life technologies A12379) and 10µg/ml 4', 6-diamidino-2-phenylindole (DAPI). Secondary antibodies for Western Blotting were purchased from Dako.

**Recombinant Proteins, Antagonists, siRNA and shRNA**

Recombinant Murine JE/CCL2 and Recombinant Murine CXCL1 were obtained from Peprotech and added to DMEM medium at indicated concentrations. CCR2 antagonist (C_28_H_34_F_3_N_5_O_4_S) and CXCR2 antagonist, Cpd-19 (C_18_H_21_N_3_O_4_) were purchased from Calbiochem. Rac1 inhibitor NSC23766 (C_24_H_38_Cl_3_N_7_) and Cdc42 inhibitor ML141 (C_22_H_21_N_3_O_3_S) were purchased from Calbiochem. Rho Inhibitor I (CT04, active site is C3 transferase) was purchased from Cytoskeleton. P38 inhibitor SB203580 (C_12_H_16_FN_3_OS), was purchased from Merck Millipore. CCL2 siRNA (m) and Control siRNA (fluorescein Conjuagate)-A were purchased from Santa Cruz Biotechnology. Silencer® Select mouse CXCL1 siRNA and Negative Control siRNA was purchased from Ambion. The CCR2 and CXCR2 Mission shRNA Bacterial Glycerol Stocks were purchased from Sigma Aldrich. The shRNA Non-Targeting (NT) vector, SHC002 was used as a negative control.

**Cell Sorting**

As described in previous studies[4], heterogeneous vascular progenitor cells which grew out from vein grafts were sorted by magnetic cell sorting kits (MACS) with anti-Sca-1 immunomagnetic microbeads (Miltenyi Biotec, GmbH, Bergisch Gladbach, Germany). Briefly, the cells were incubated with the antibody-conjugated/coated microbeads at 4°C and then selected using a magnetic cell separator (Miltenyi Biotec). Sca-1 positive vascular progenitor cell populations were expanded for up to 5 population doublings.

**Immunofluorescence Staining**

Cells were fixed with 4% paraformaldehyde, permeabilized with 0.1% Triton X-100 (Sigma) and blocked with 5% normal donkey serum (Dako) for 1 hour at room temperature. Incubation of cells with primary antibodies was performed at 4℃ overnight, followed by incubation with secondary antibodies (Corresponding fluorescent-conjugated IgG antibodies were used as secondary antibodies (Invitrogen)) for 45 mins at 37℃. Then cells were counterstained with DAPI (Sigma) for 5 mins at room temperature and mounted with fluorescent mounting media (Dako). Images were acquired using an Olympus IX81 microscope and Volocity software (PerkinElmer).

**Transwell Chemotaxis Assay**

Migration assays were performed using transwell inserts with 8.0 µm pore membrane filters (Corning). Vascular progenitor cells were loaded onto the upper chamber at 5x10^4^ cells in serum free media, while the bottom chamber contained SMC conditional medium or serum free medium with indicated concentrations of recombinant murine CCL2 or CXCL1. Serum free medium served as negative control. After an overnight incubation, non-migrating cells on the upper side of the filters were carefully washed and removed using a swab. Vascular progenitor cells on the underside of the membrane were fixed with 4% PFA for 10 mins followed by 0.1% crystal violet solution staining at room temperature for 15 mins. Data was expressed as the fold of migrated vascular progenitor cells compared to the control in 9 fields of each insert (at 20x). For experiments which involved inhibitors, vascular progenitor cells were pre-treated with the respective inhibitors before transferring to transwells.

**Scratch-wound Assay**

Vascular progenitor cells were seeded in a 12-well plate. Upon reaching complete confluency, a straight scratch was made using a pipette tip to stimulate a 'wound' through the middle of each well. The wells were gently washed with PBS to remove cell debris prior to treatment with SMC conditional medium or serum free medium with or without (control) recombinant murine CCL2 or CXCL1. After an overnight incubation, the migration of vascular progenitor cells into the “wound” area was quantified as the mean number of the cells which migrated into the “wound” in 5 random fields of each well using a phase contrast microscope(at 10x).

**Time-lapse Microscopy**

Cells were seeded onto 6-well plates and held with or without SMC conditioned media at 37˚C and 5% CO_2_ on the stage of a fully motorized, multi-field time-lapse microscope (Eclipse TE 2000-E; Nikon) with a charge-coupled device camera (ORCA; Hamamatsu Photonics). Bright-field images were acquired for 20 hours with pictures taken at 5-min intervals using the 10x objective. Images were acquired using Volocity software (PerkinElmer), and cells were tracked using ImageJ software (National Institute of Health). Analysis of cell speed and persistence was performed using the Chemotaxis plug in from Integrated BioDiagnostics.

**RT-PCR**

Total RNA was isolated from vascular progenitor cells using a QIAGEN RNeasy Mini kit according to the manufacturer’s instructions. 1 μg RNA was reverse transcribed into cDNA with random primers using the MMLV reverse transcriptase kit (Promega). PCR was carried out on 50ng cDNA to amplify the mouse CCL2 receptor CCR2 gene using primers:

Forward 5'- TCCACTCTACTCCCTGGTATTC-3';

Reverse 5' - TGGCCAAGTTGAGCAGATAG - 3'.

The murine CXCL1 receptor CXCR2 gene primers:

Forward 5’-TCTGGCCCTGCCCATCTTAATTCT-3’;

Reverse 5’- AAAGTCTGAGGCAGGATACGCAGT -3’.

The PCR products were analyzed on 2% agarose gels and assessed using a BioSpectrum AC Imaging System and Vision- WorksLS software.

**Quantitative Real Time Polymerase Chain Reaction (qPCR)**

The methods described above were used for RNA extraction and cDNA reverse transcription. Real time RT-PCR was performed using 20ng of cDNA per sample with a SYBR Green Master Mix in a 20 μl reaction. Ct values were measured using the Eppendorf Mastercycler ep Realplex and GAPDH was used as an endogenous control to normalize the amounts of RNA in each sample. The sequences of chemokine CCL2 primer sets are:

Forward 5’-AGTAGGCTGGAGAGCTACAA-3’,

Reverse 5’-GTATGTCTGGACCCATTCCTTC-3’,

The sequences of chemokine CXCL1 primer sets are:

Forward 5’-GCTGGGATTCACCTCAAGAA-3’,

Reverse 5’-TGGCTATGACTTCGGTTTGG-3’,

**Western Blot Analysis**

Harvested vascular progenitor cells were lysed with lysis buffer (25 mM Tris-HCl pH 7.5, 150 mM NaCl, 1 mM EDTA pH 8.0, 1%Triton X-100 plus protease inhibitors(Roche), PhosSTOP Phosphatase Inhibitor Cocktail Tablets (Roche)) and proteins were sequentially measured using the Bradford method. 40 μg of protein lysate was applied to 4-12% Bis-Tris Protein gels (NuPAPE, Novex) before being transferred to a nitrocellulose membrane (Amersham Biosciences), followed by a standard western blotting procedure.

**CCL2 and CXCL1 gene knockdown**

Gene suppression of CCL2 or/and CXCL1 in mouse smooth muscle cells was carried out using the Basic SMC Nucleofector® Kit (Lonza) with CCL2 siRNA (Santa Cruz Biotechnology) or/and CXCL1 siRNA (ambion) as the manufacturers described. Control siRNA (FITC Conjugate)-A (Santa Cruz Biotechnology) or Negative control siRNA (ambion) was used as a negative control. After gene ablation, the level of down regulation of total RNA or proteins was assessed using real time qPCR or ELISA analysis, respectively.

**CCR2 and CXCR2 gene knockdown**

Gene ablation of CCR2 or/and CXCR2 was carried out using short hairpin RNA (shRNA) lentiviral plasmid transfer as previously described[5, 6][_ENREF_56](#_ENREF_56). A non-targeting vector (SHC002) was used as a negative control. Total gene and protein levels were assessed using qPCR and flow cytometry analysis after gene ablation.

**FACS analysis**

Vascular progenitor cells were treated with/without SMC conditioned media, and incubated with antibodies (mouse anti-CXCR2 PE-conjugated antibody (R&D), rabbit anti-CCR2 (abcam) or mouse IgG PE (BD biosciences) for 30 mins on ice before analysis by FACS in order to test the change of CXCR2 or CCR2 receptor expression levels. Data analysis was carried out using FlowJo software.

**Femoral artery injury**

Mice were anesthetized with (ketamine and medetomidine hydrochloride) and the surgical procedure was similar to that described previously[_ENREF_57](#_ENREF_57)[7]. Both of the femoral arteries of each mouse were injured by inserting a 0.25 mm guide wire (CROSS-IT 100XT, HI-TORQUE) 4-mm length from one of the distal muscle branches to femoral artery. One artery was seeded with Sca-1^+^ vascular progenitor cells (1x10^6^ cells) within 25 μl Matrigel® Basement Membrane Matrix (Corning), and the other injured artery served as a control. Arteries were harvested at day 7 or day 14 and used to prepare frozen or paraffin sections.

For the siRNA knockdown experiments *in vivo*, either 5 μg CXCL1 or Negative Control siRNA dissolved in 30% pluronic Gel-127 was perivascularly delivered to the femoral arteries immediately after wire injury. After 3 days, GFP-Sca1^+^ vascular progenitor cells (1x10^6^ cells) within 25 μl Matrigel® Basement Membrane Matrix were seeded on the adventitia of both siRNA treated femoral arteries. Arteries were harvested for either RNA extraction or *en face* staining after a further 72 hours.

***En face* staining**

*En face* staining was used for quantification of reendothelializaiton. Briefly, femoral arteries were fixed with 4% paraformaldehyde, then permeabilized and blocked with a solution of 0.5% Triton X-100 and 5% donkey serum in PBS. Incubation of vessels with primary antibodies was performed at 4℃ overnight, followed by incubation with corresponding fluorescent-conjugated secondary antibodies (Invitrogen) or IgG as negative control for 2 hours at room temperature. Then nuclei were stained with DAPI for 5 mins at room temperature before vessel segments were mounted with the endothelium face up on a glass slide. Images were taken using a Leica SP5 confocal microscope and assessed using LAS AF lite software

**Creation of chimeric mice**

The procedure used for creating chimeric mice was similar to previously described[8]. In brief, bone marrow transplantation was carried out on the CCL2^+/+^ mice and CCL2^-/-^ mice separately. Bone marrow cells were obtained from the femurs and tibias of either CCL2^+/+^ or CCL2^-/-^ mice (donors) and injected (1x10^7^cells in 0.2ml) into the tail veins of the 6-8 week old CCL2^-/-^ mice or CCL2^+/+^ mice (recipients) which received lethal irradiation (950 Rads) before. Femoral artery injury was performed 3 weeks after bone marrow transplantation.

**Rac1/Cdc42 GTPase activation assay**

The activation assay of GTP-bound Rac1/Cdc42 was carried out according to the manufacturer’s instructions (Rac1/Cdc42 Activation Assay Kit (Upstate, Millipore)). In addition to the solutions from the kit, PAK-PBD beads from cytoskeleton, Protease Inhibitor Cocktail (Sigma-Aldrich) and PhosSTOP Phosphatase Inhibitor Cocktail Tablets (Roche) were also used. Briefly, cells were stimulated with the indicated treatments then placed on ice and scraped within 1x MLB (25 mM HEPES, pH 7.5, 150 mM NaCl, 1% Igepal CA-630, 10 mM MgCl2, 1mM EDTA and 2% glycerol), and samples were centrifuged for 10 mins. Aliquots were collected for total Rac1 or Cdc42 (input) and GTPγS (positive) and GDP (negative) control analysis. Lysates were rotated with 20µg of PAK-PBD agarose beads at 4 °C for 1 hour. The agarose pellet was washed twice and re-suspended in 25µl SDS sample buffer. Samples were separated by 4–12% Bis-Tris gels, transferred to nitrocellulose membranes, and blotted for Rac1 or Cdc42 antibodies (Upstate, Millipore).

**RT^2^ Profiler^TM^ PCR Arrays for Mouse Chemokines and Receptors**

Total RNA (0.5 μg) (extracted from either mouse peritoneal macrophages, vascular smooth muscle cells, Sca-1^+^ vascular progenitor cells or one cloned colony of vascular progenitor cells cultured *in vitro)* was reverse transcribed to cDNA and used to screen mouse chemokine receptors using quantitative real-time PCR arrays according to the manufacturer's instructions (Qiagen PAMM-022Z). Reactions were performed in an Eppendorf® Mastercycler® ep Realplex model 4S. Acquired data were analyzed using the 2^-(averageΔCT)^ method to determine the expression level of each transcript normalized to the expression level of housekeeping gene controls.

**SUPPLEMENTAL REFERNCES**

1. Hu Y, Zhang Z, Torsney E et al. Abundant progenitor cells in the adventitia contribute to atherosclerosis of vein grafts in ApoE-deficient mice. **The Journal of clinical investigation***.* 2004;113:1258-1265.

2. Corning Incorporated Life Sciences. Cell Cloing by Serial Dilution in 96 Well Plates Protocol. Available at http://www.level.com.tw/html/ezcatfiles/vipweb20/img/img /34963/3-2Single_cell_cloning_protocol.pdf (http://www.level.com.tw/html/ezcatfiles /vipweb20/img/img/34963/3-2Single_cell_cloning_protocol.pdf) Accessed March 28, 2016

3. Hu Y, Zou Y, Dietrich H et al. Inhibition of neointima hyperplasia of mouse vein grafts by locally applied suramin. **Circulation***.* 1999;100:861-868.

4. Xiao Q, Zeng L, Zhang Z et al. Sca-1+ progenitors derived from embryonic stem cells differentiate into endothelial cells capable of vascular repair after arterial injury. **Arteriosclerosis, thrombosis, and vascular biology***.* 2006;26:2244-2251.

5. Shea-Donohue T, Thomas K, Cody MJ et al. Mice deficient in the CXCR2 ligand, CXCL1 (KC/GRO-alpha), exhibit increased susceptibility to dextran sodium sulfate (DSS)-induced colitis. **Innate immunity***.* 2008;14:117-124.

6. Margariti A, Winkler B, Karamariti E et al. Direct reprogramming of fibroblasts into endothelial cells capable of angiogenesis and reendothelialization in tissue-engineered vessels. **Proceedings of the National Academy of Sciences of the United States of America***.* 2012;109:13793-13798.

7. Zeng L, Xiao Q, Margariti A et al. HDAC3 is crucial in shear- and VEGF-induced stem cell differentiation toward endothelial cells. **The Journal of cell biology***.* 2006;174:1059-1069.

8. Coussens LM, Tinkle CL, Hanahan D et al. MMP-9 supplied by bone marrow-derived cells contributes to skin carcinogenesis. **Cell***.* 2000;103:481-490.

**Supplement Figure Legend**

***
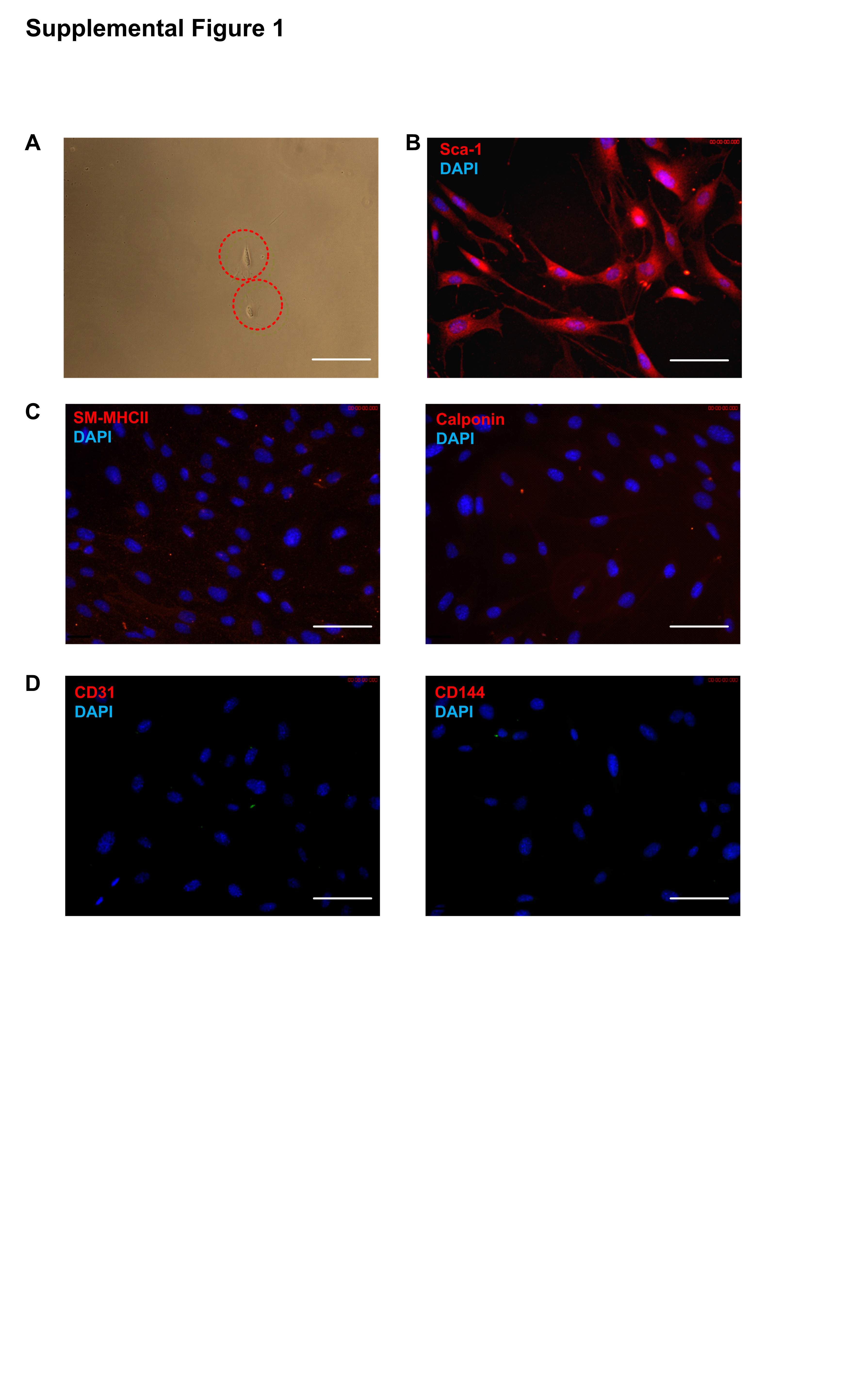
***

Figure 1. **Isolation of clones and characterization.** Single cells were obtained by serial dilution, approximately 3% to 5% of cells formed clones that could be gradually expanded. **A,** Two cells (in red circles) from the same clone colony are shown in the represented image. **B, C, D.** Clone colonies were characterized using immunofluorescence staining for progenitor cell marker (Sca-1), SMC markers (SM-MHCII, Calponin) or endothelial cell markers (CD31，CD144). Scale bars, 100µm. SM-MHCII, smooth muscle cell myosin heavy chain class II.

**
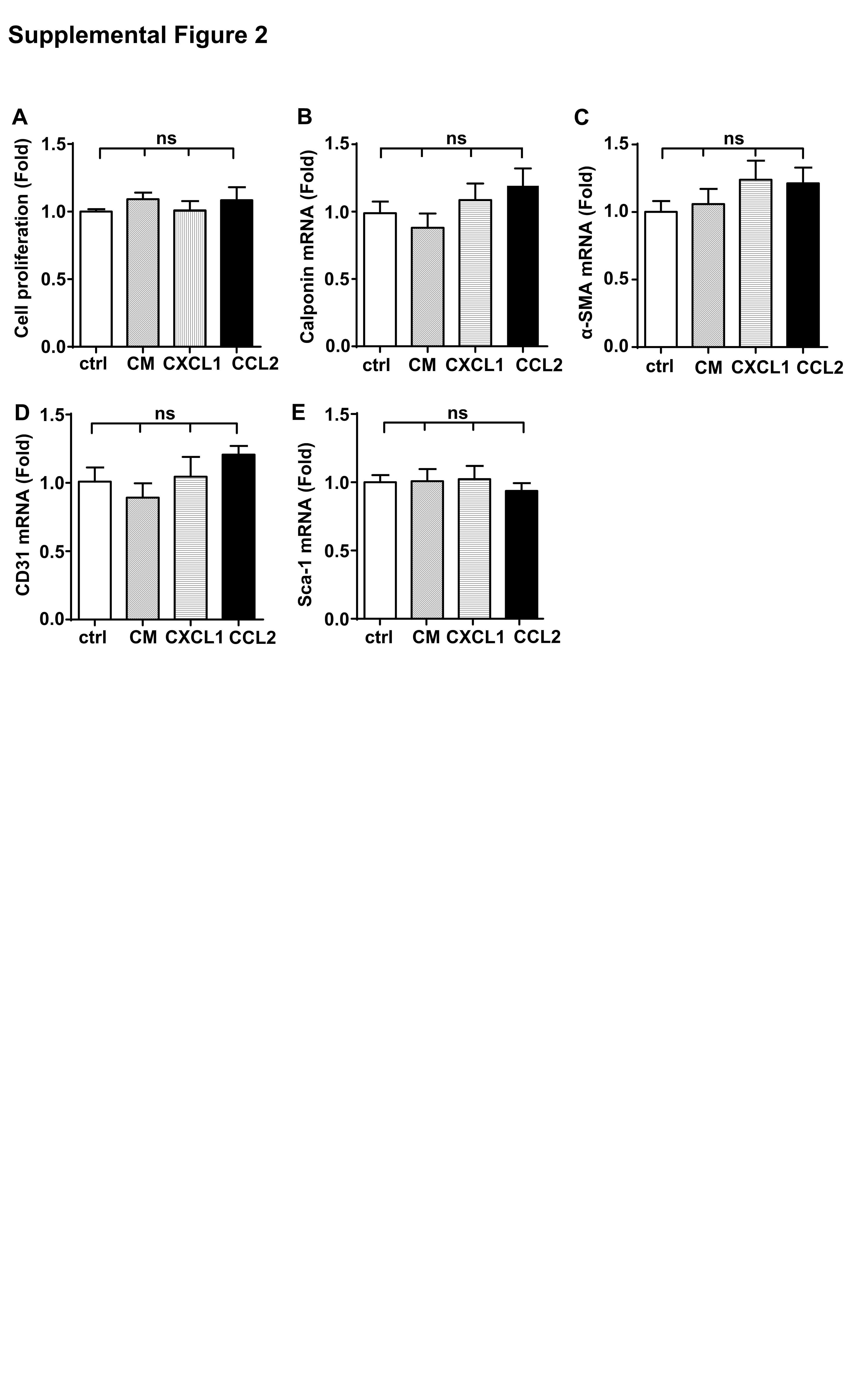
**

Figure 2. **SMC-CM has no effect on VPCs proliferation and differentiation****.**

**A.** Evaluation of vascular progenitor cell proliferation in response to SMC conditioned medium, CXCL1or CCL2 treatments compared to an untreated control after 18 hours was by BrdU incorporation assay. **B, C, D, E.** Total RNA from vascular progenitor cells was harvested and subjected to qPCR for analysis of SMC markers (α-SMA, SM-MHCII), endothelial cells marker (CD31) and progenitor cells marker (Sca-1) expression. All graphs are shown as mean ± SEM of n=3. ns, P>0.05. ctrl, control, serum free medium. CM, SMC conditioned medium.


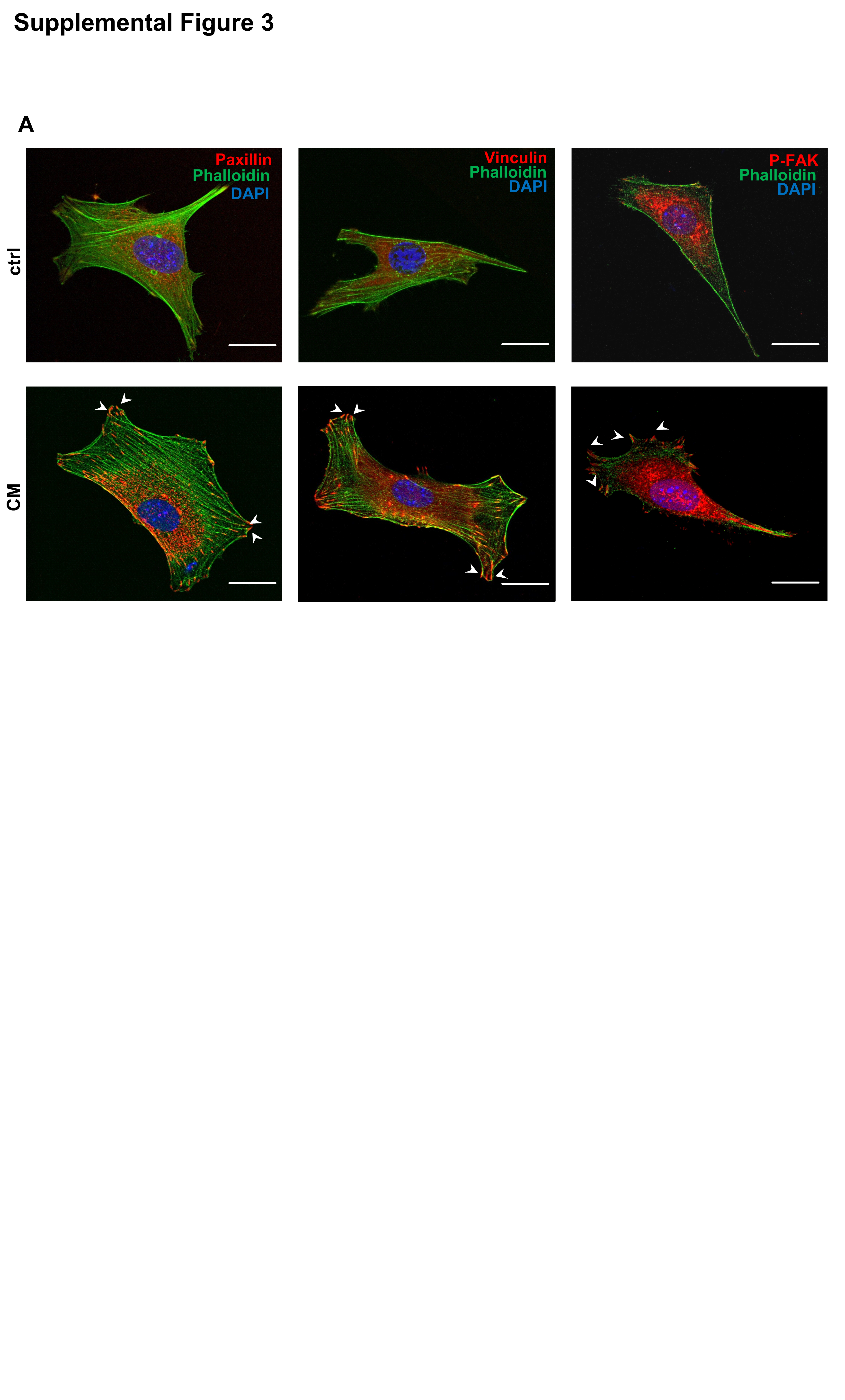


Figure 3. **SMC conditioned medium increases paxillin, vinculin and phosphorylated FAK expression. A.** Vascular progenitor cells were treated with SMC conditioned medium for 5 minutes before immunofluorescence staining for paxillin, vinculin and phosphorylated FAK. Scale bars, 25µm. ctrl, control, serum free medium. CM, SMC conditioned medium. p-FAK, phosphorylated FAK.


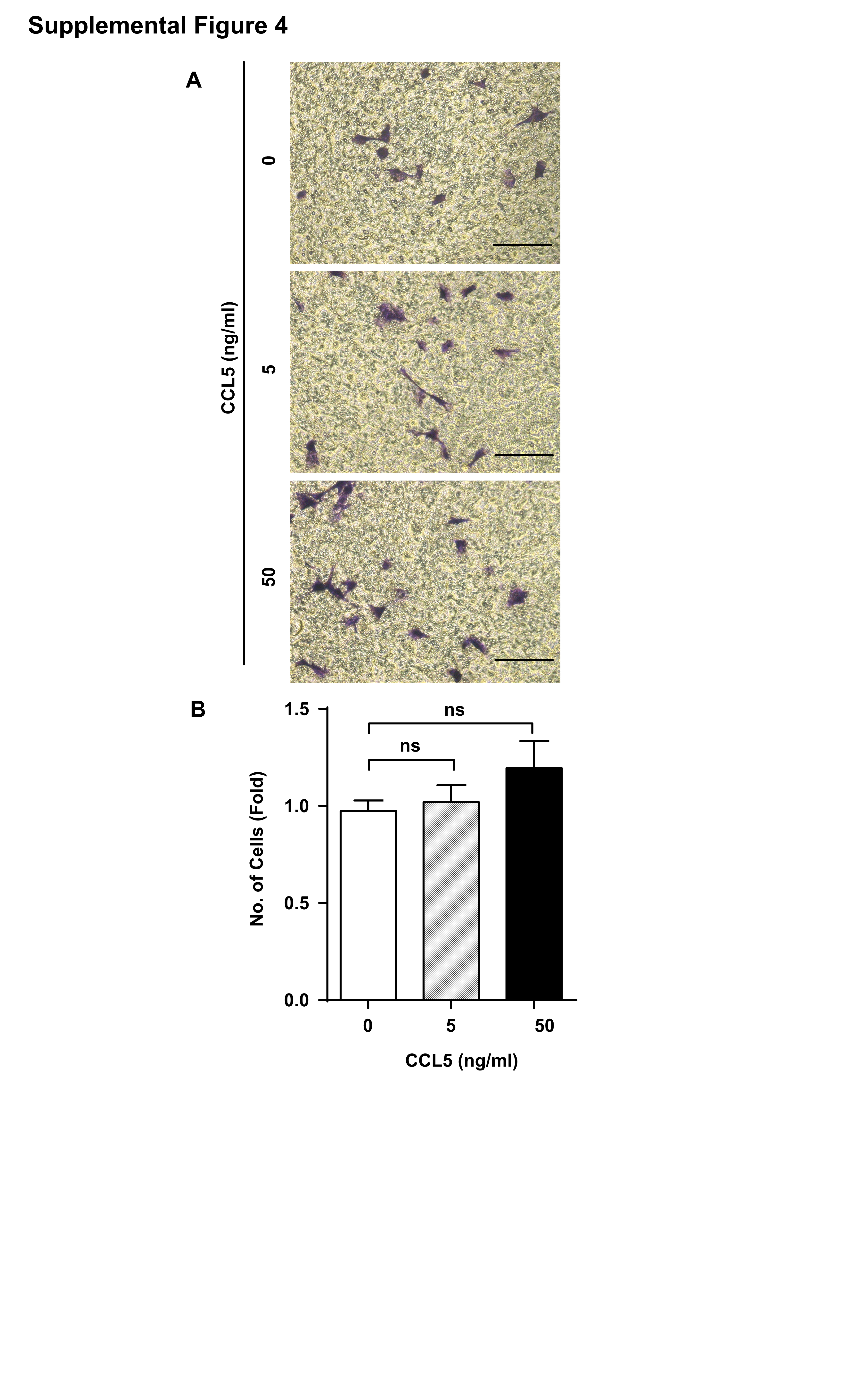


Figure 4. **CCL5 has no effect on VPCs migration. A.** The transwell assay was performed on vascular progenitor cells that migrated toward either serum free medium (control) or serum free media containing murine recombinant CCL5 (5ng/ml, 50ng/ml). Transwell migration assays show VPCs migrated towards serum free medium with or without mouse recombinant CCL5 (5ng/ml, 50ng/ml). Scale bars, 100µm. **B.** The graph is shown as mean ± SEM of n=3. ns, P>0.05.


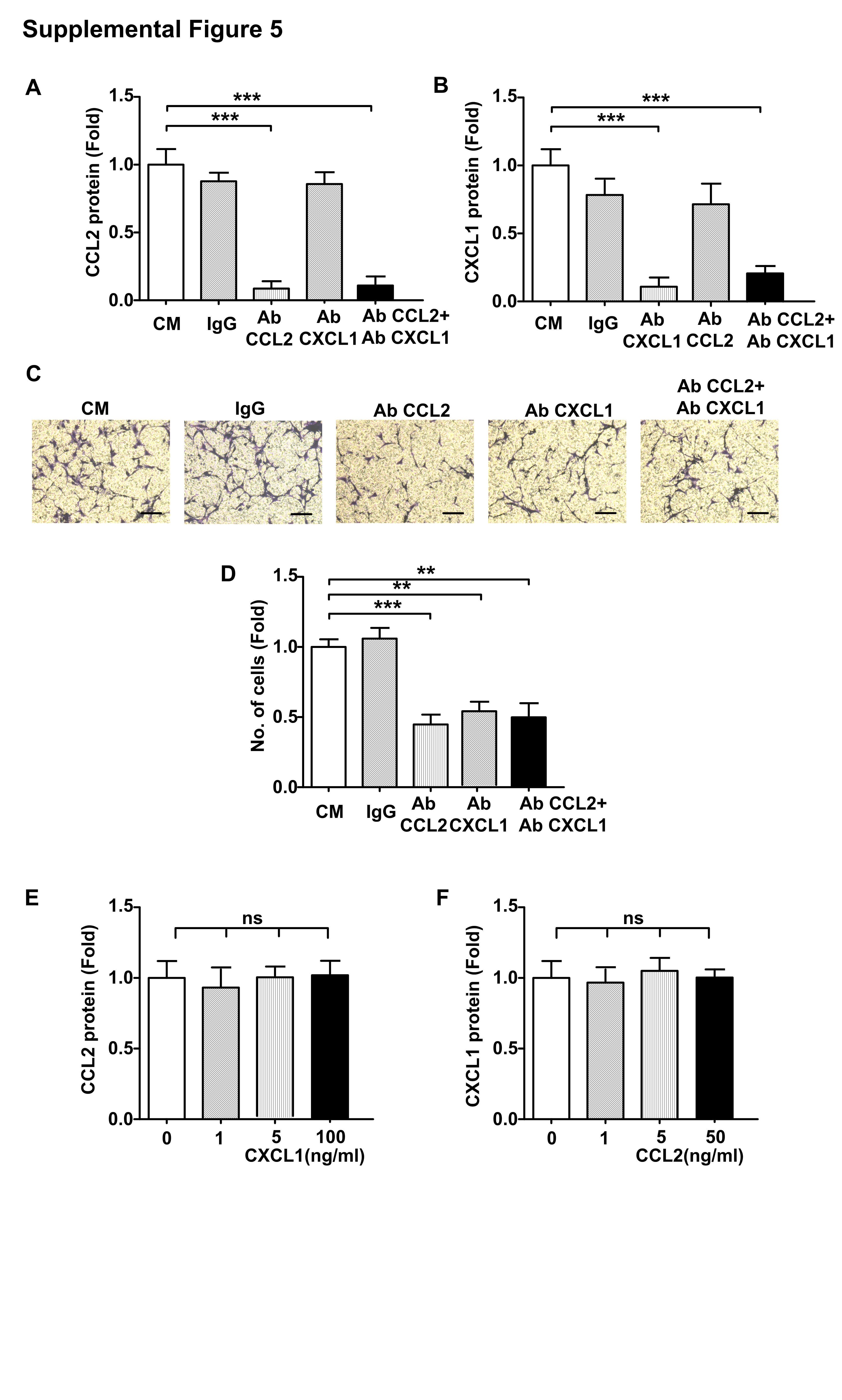


Figure 5. **The effects of CCL2 and CXCL1 are neither cumulative nor redundant in mediating VPC migration. A-D.** The SMC conditioned medium was treated with either neutralizing antibodies for CCL2 and/or CXCL1 or the relevant control IgG for 1 hour at 37℃ before pull-down of chemokines combined with antibodies using protein G beads. The supernatant was collected and used to measure the concentrations of CCL2 (**A**) and CXCL1 (**B**) using corresponding ELISA kits. The transwell assay (**C, D**) was performed on VPCs that migrated toward SMC conditioned medium (positive control) or SMC conditioned medium after depletion of chemokines with IgG, CCL2 and/or CXCL1 neutralizing antibodies pull down. Scale bars, 100µm. **E, F.** The VPCs were treated with either CXCL1 or CCL2 at indicated concentrations overnight followed by measurement of concentrations of CCL2 and CXCL1 using ELISA. The graphs are shown as mean±SEM of n=3, **p<0.01, ***p<0.001, ns, P>0.05.


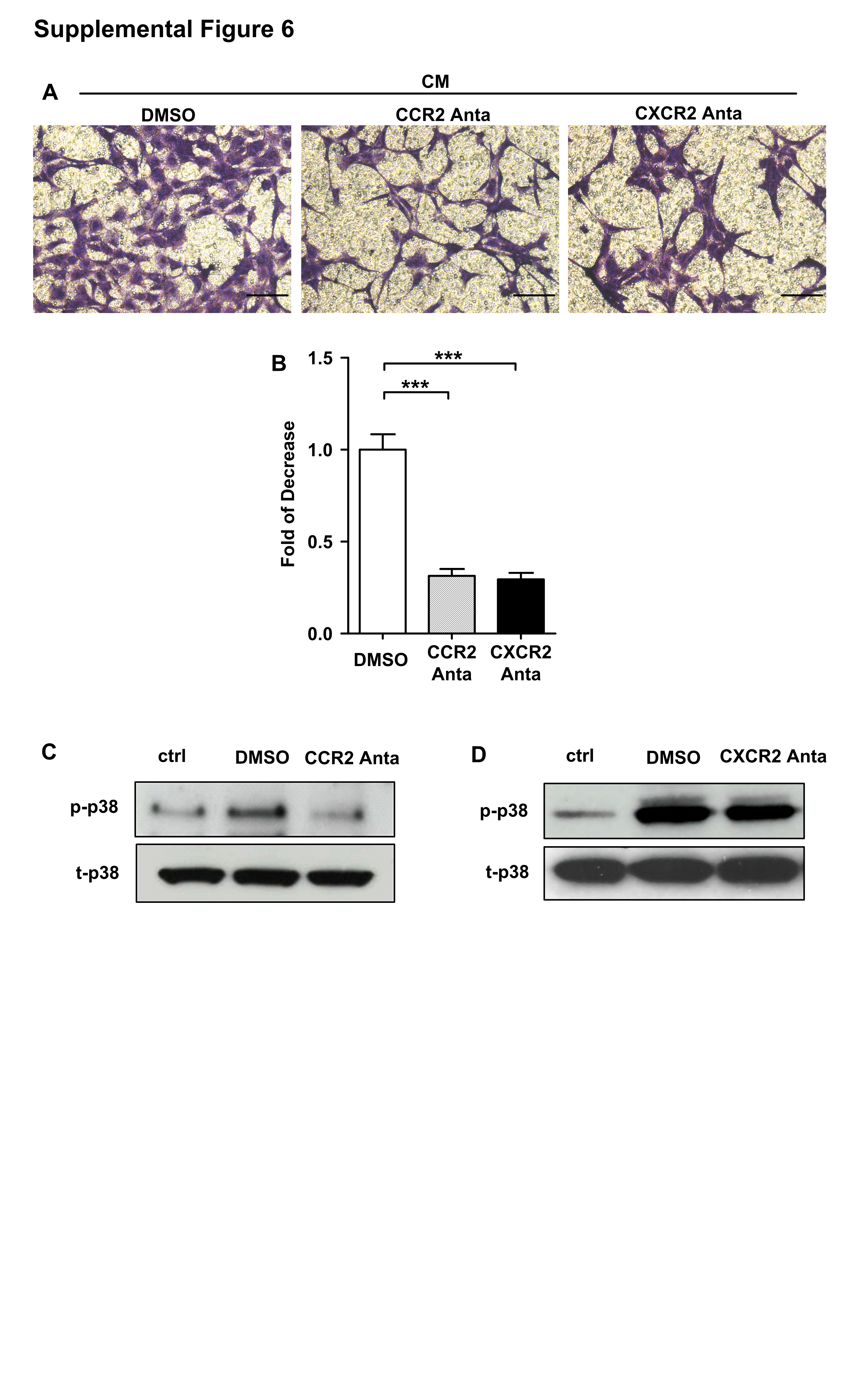


Figure 6. **Antagonists of CCR2 or CXCR2 inhibited SMC-mediated VPC migration via the p38 signaling pathway.** **A, B**. The transwell assay was performed on vascular progenitor cells that were pre-treated with DMSO, antagonists of CCR2 or CXCR2 1 hour before migrating toward SMC conditioned medium. Scale bars, 100µm. **C, D**. Western blotting was performed on vascular progenitor cell lysates (40 μg per condition) to detect phosphorylation of p38 and total p38. Untreated cells served as the control. The quantification is represented as mean ± SEM of n=3. ***p<0.001. ctrl, control, serum free medium. CM, SMC conditioned medium. DMSO, Dimethyl sulfoxide. CCR2 Anta, CCR2 antagonist. CXCR2 Anta, CXCR2 antagonist.


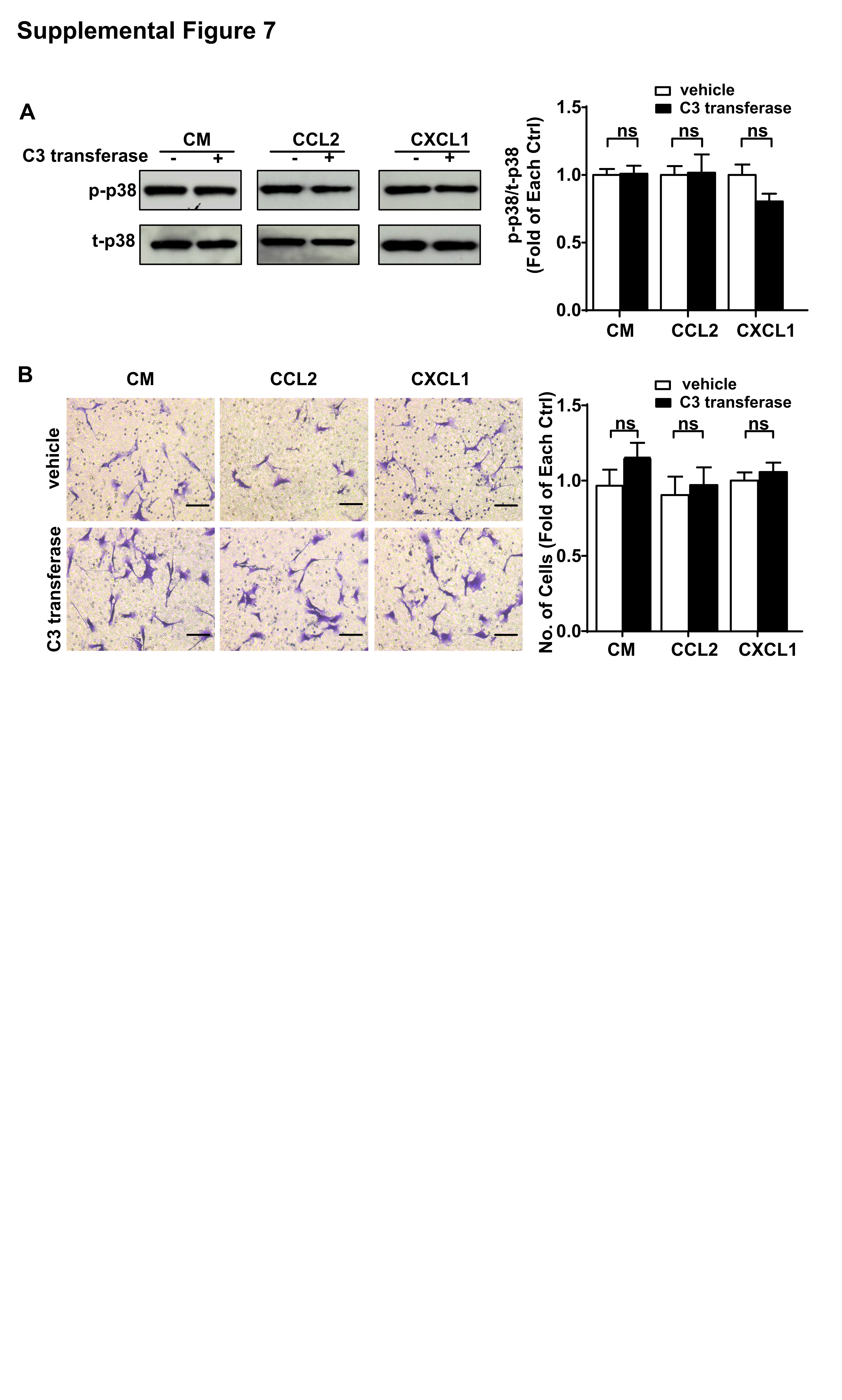


Figure 7. **The RhoA signalling pathway is not involved in the SMC induced VPCs migration. A.** After pre-treatment with either vehicle or C3 transferase (2µg/ml) for 2 hours, VPC were stimulated with SMC conditioned medium or recombinant CCL2 or CXCL1 before cell lysates were harvested for western blotting to detect the p-p38 and t-p38. **B.** The transwell assay was performed on VPCs that were pre-treated with either vehicle or C3 transferase (an inhibitor of RhoA) (2µg/ml) for 2 hours before migration toward either SMC conditioned medium or recombinant CCL2 or CXCL1. Scale bars, 100µm. Quantification is represented as mean ± SEM of n=3. ns, P>0.05. CM, SMC conditioned medium. vehicle, sterile distilled water.


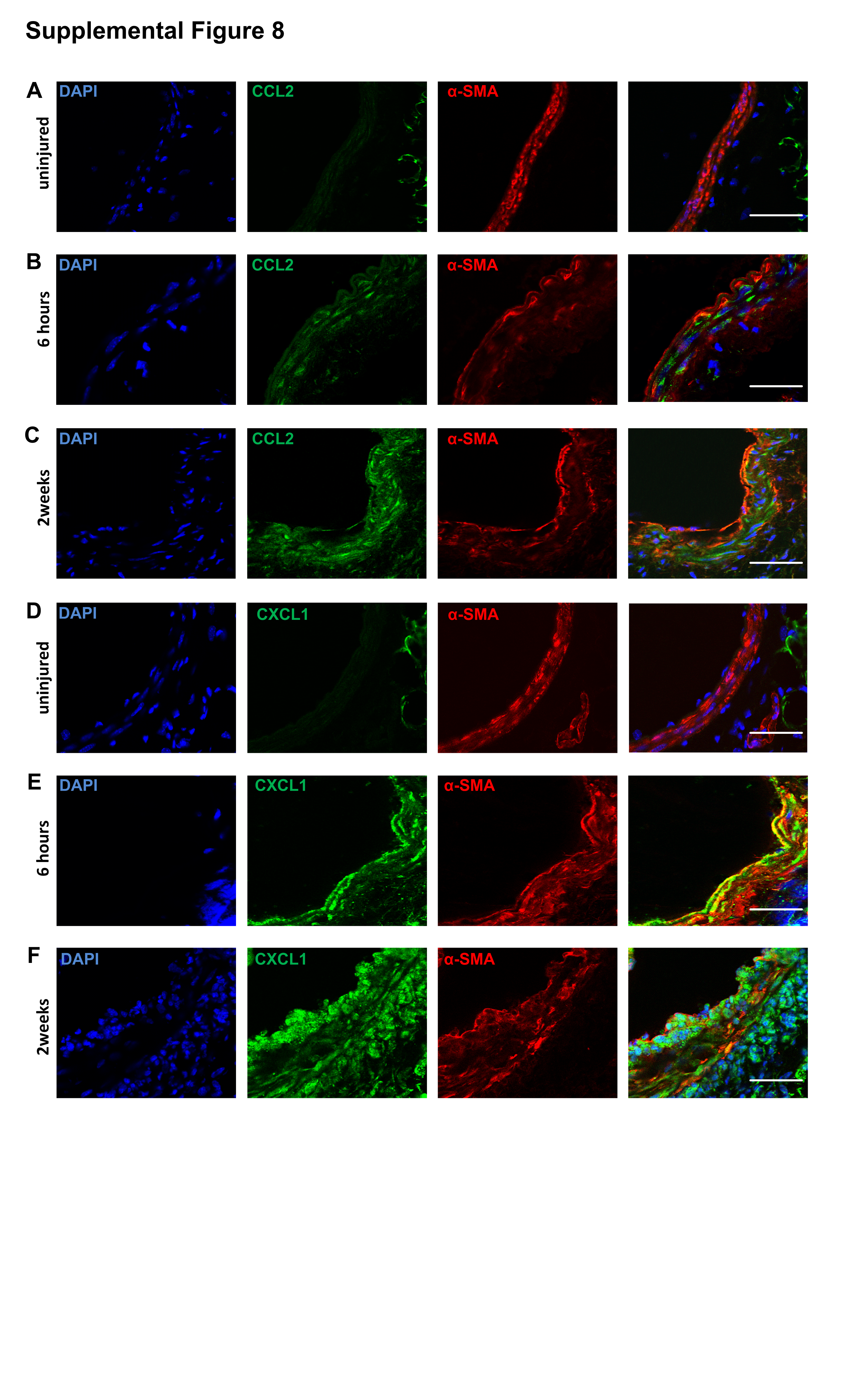


Figure 8. **CCL2 and CXCL1 are expressed on wire-injured vessels *in vivo*.** Femoral arteries of C57BL/6J mice were harvested after wire injury or from uninjured animals. Frozen sections of intact vessels or vessels 6 hours or 2 weeks after wire injury were co-stained with immunofluorescent markers either CCL2 (**A, B, C**) or CXCL1(**D, E, F**) and α-SMA. n=4 mice/group. Scale bars, 50µm.


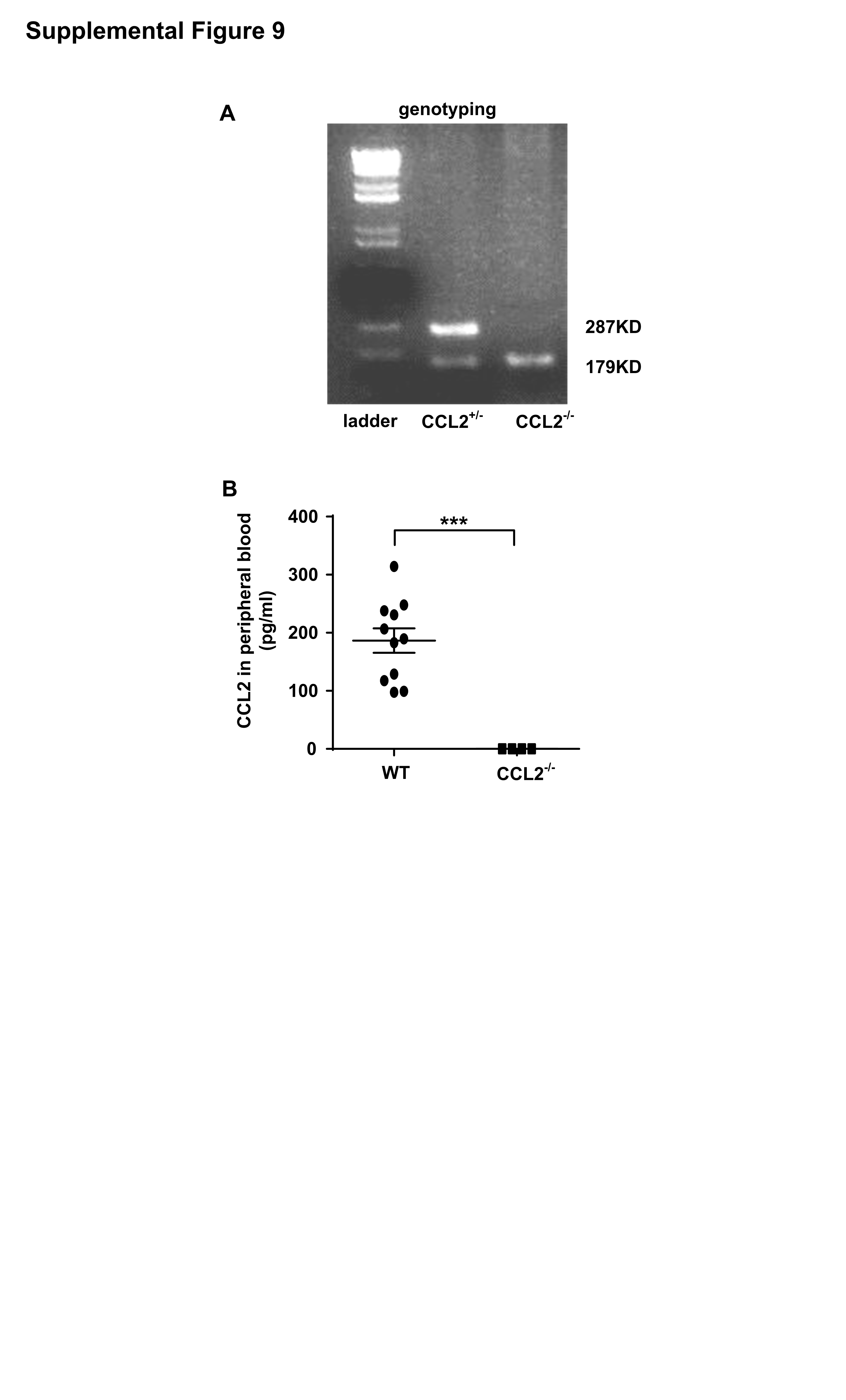


Figure 9. **Identify the CCL2^-/-^ mouse genotype and CCL2 level in the peripheral blood. A.** To expand CCL2^-/-^ mice number, CCL2^-/-^ mice were crossed with CCL2^+/+^. A piece of ear tissue from each mouse was used to genotype offspring. The image shows a heterozygote (CCL2^+/-^) with bands at 287bp and 179bp and a mutant (CCL2^-/-^) with only one band at 179bp. **B.** Peripheral blood was collected from the vena cava before euthanasia of WT or CCL2^-/-^ mice and CCL2 level was measured using a murine CCL2 Quantikine ELISA kit from serum of each sample. The graph is shown as mean ± SEM of n=11 mice/group, ***p<0.001.


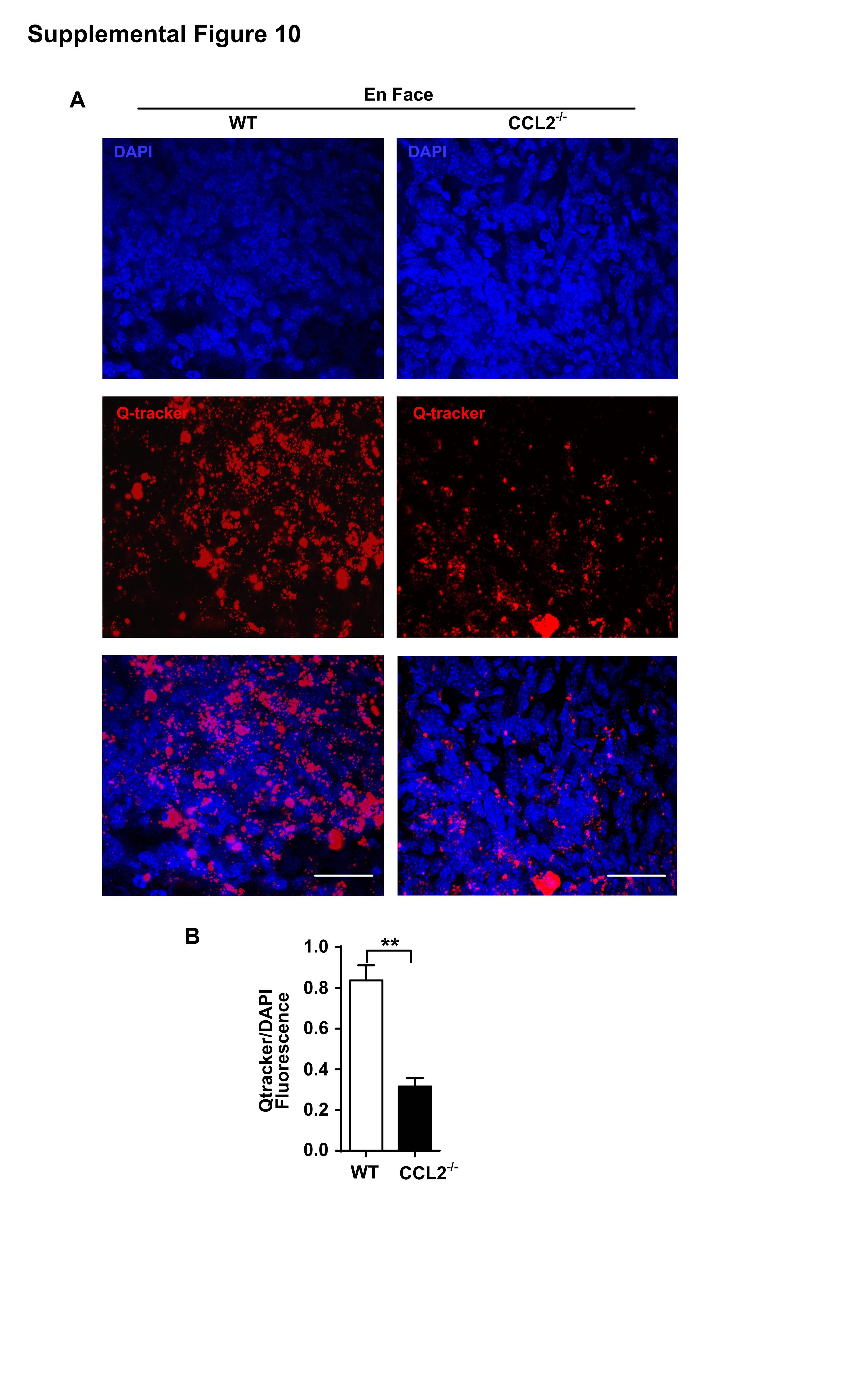


Figure 10. **Lack of CCL2 inhibits Sca-1^+^ vascular progenitor cell migration *in vivo*.**

**A.** Sca-1^+^ vascular progenitor cells (1x10^6^), which were labeled with Q-tracker to locate them as they migrated, were seeded in the adventitia of each injured femoral artery. 72hrs post injury, the vessels were harvested. *En face* staining shows that cells migrated to the intima side of the vessels of WT and CCL2^-/-^ mice. Scale bars, 25µm. **B.** The ratio of the fluorescence intensity (Qtracker:DAPI) is represented in the graphs. The graph is shown as mean ± SEM of n=8 mice/group, **p<0.01.


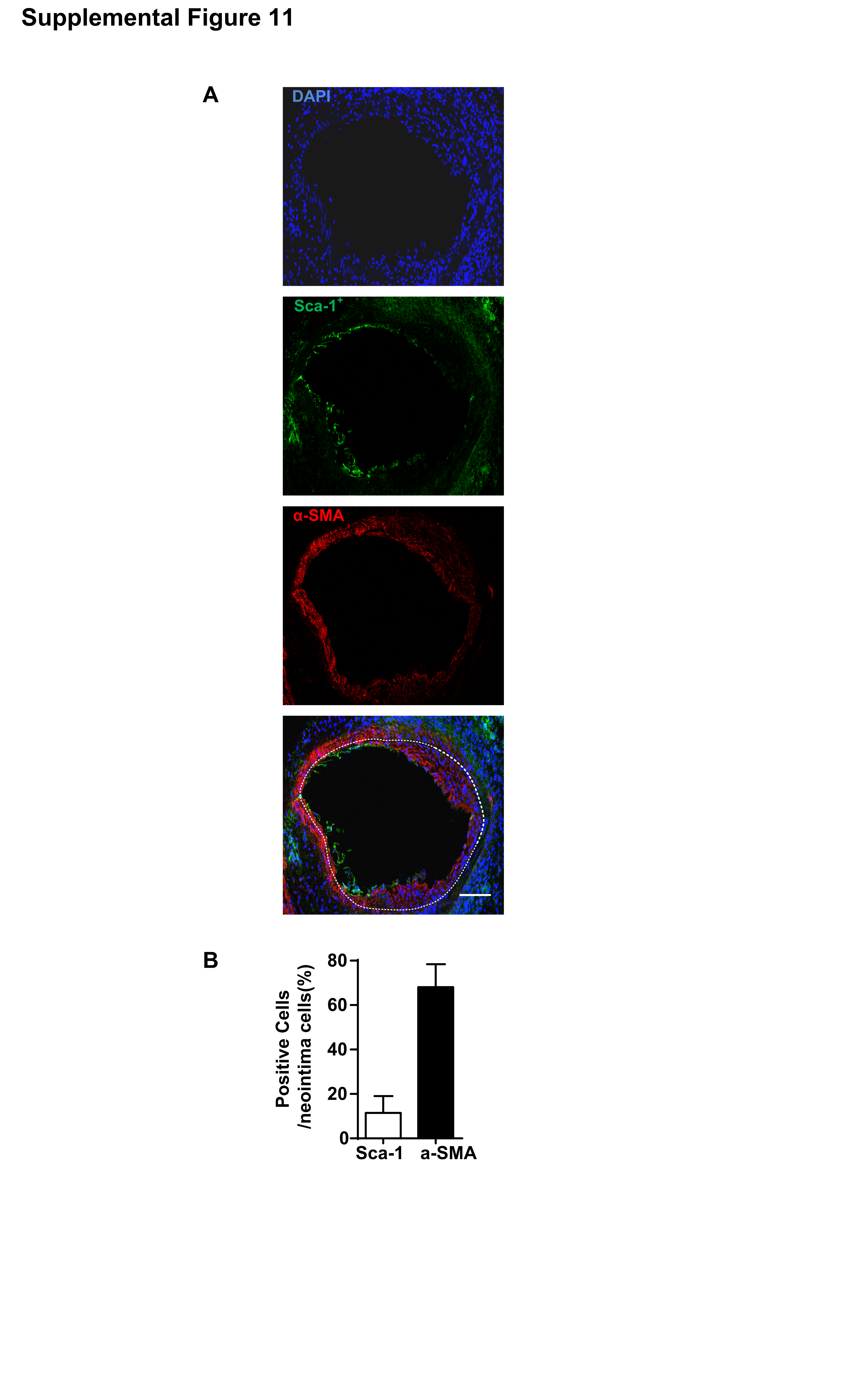


Figure 11. **Characterization of cells in the neointima.** **A.** Femoral arteries without seeding VPC in the adventitia were harvested 2 weeks after wire injury from C57BL/6J mice and stained with Sca-1 and α-SMA (white dotted line indicates internal elastin, and the circled part is neointima area). Scale bars, 50µm. **B.** The ratios of positively stained cells with each marker in the neointima area are quantified and shown in the graph.


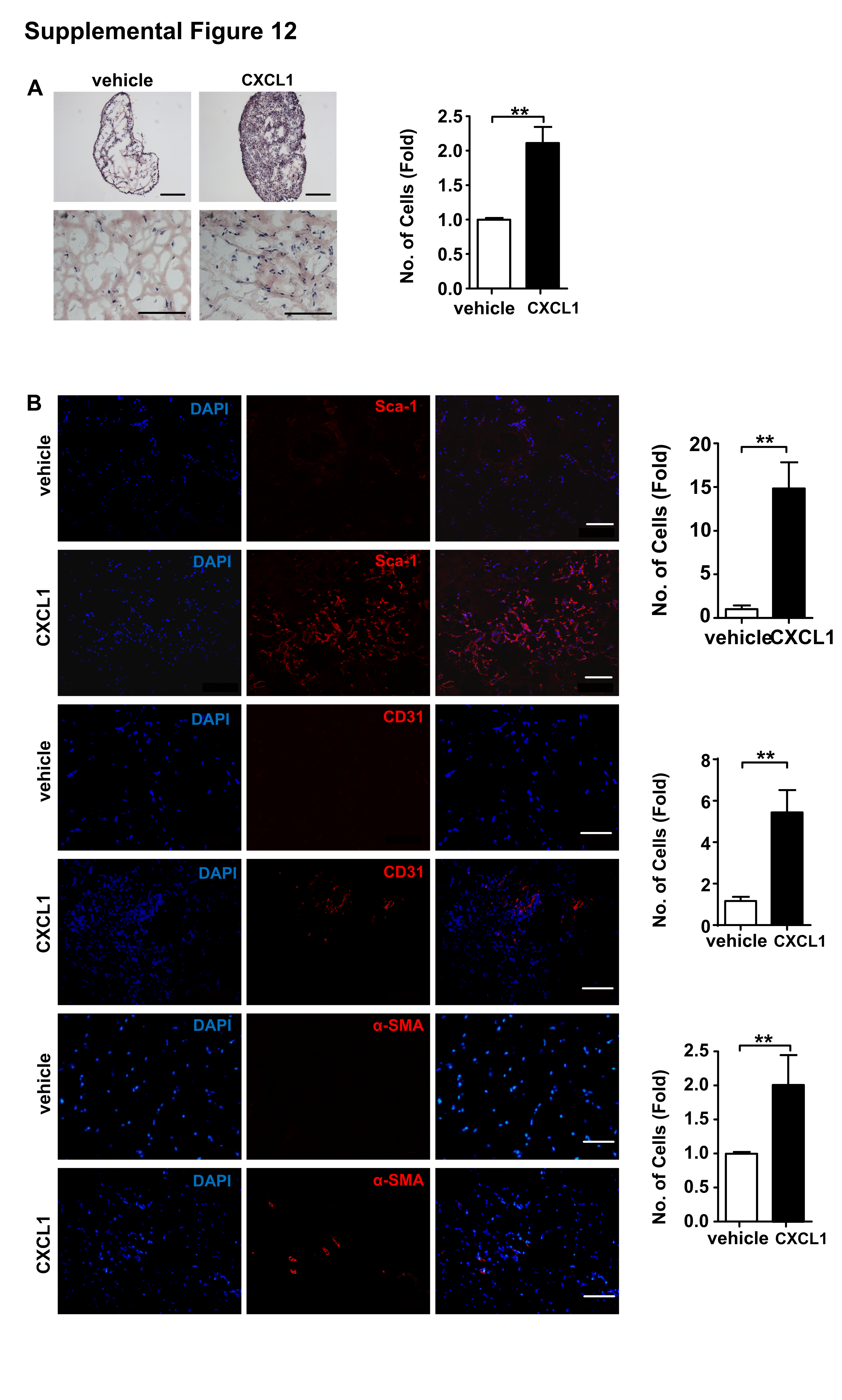


Figure 12. **CXCL1 can induce Sca-1 positive cell migration *in vivo*.** Using a matrigel plug assay, matrigel including PBS or mouse recombinant CXCL1 was injected subcutaneously into C57BL/6J mice. The matrigel plugs were harvested 2 weeks later and the frozen sections were stained with either (A) hematoxylin–eosin (Scale bars, 100µm) or (B) immunofluorescent markers (Sca-1, CD31, α-SMA) (Scale bars, 100µm). The numbers of cells migrated into the matrigel (quantification from bottom images in A) or positively stained with each marker are quantified and shown in the graphs. Graphs are shown as mean±SEM of n=8, **P<0.01. vehicle, sterile distilled water.


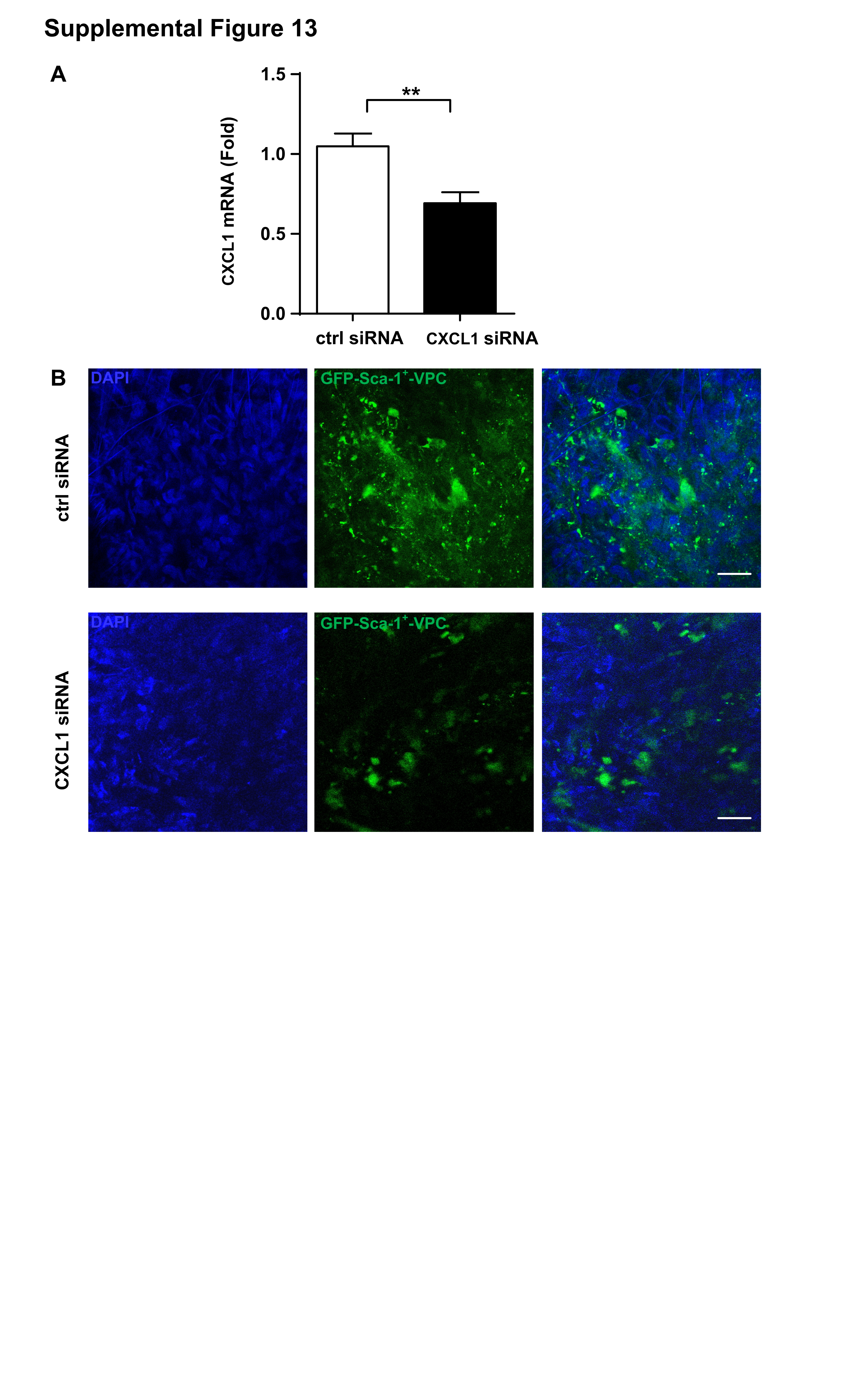


Figure 13. **CXCL1 small interfering RNA inhibits Sca-1^+^ cells migration *in vivo*.**

**A,** Quantification of CXCL1 mRNA level 6 days after perivascular application of CXCL1 or negative control siRNA dissolved in pluronic gel by qPCR. Graphs are shown as mean±SEM of n=4, **P<0.01. **B.** Representative images of *En face* staining show the cells migrated to the intima side of the vessels 72hrs post seeding GFP-Sca-1^+^-VPC (1x10^6^) in the adventitia of femoral arteries treated with CXCL1 or control siRNA. Scale bars, 25µm.


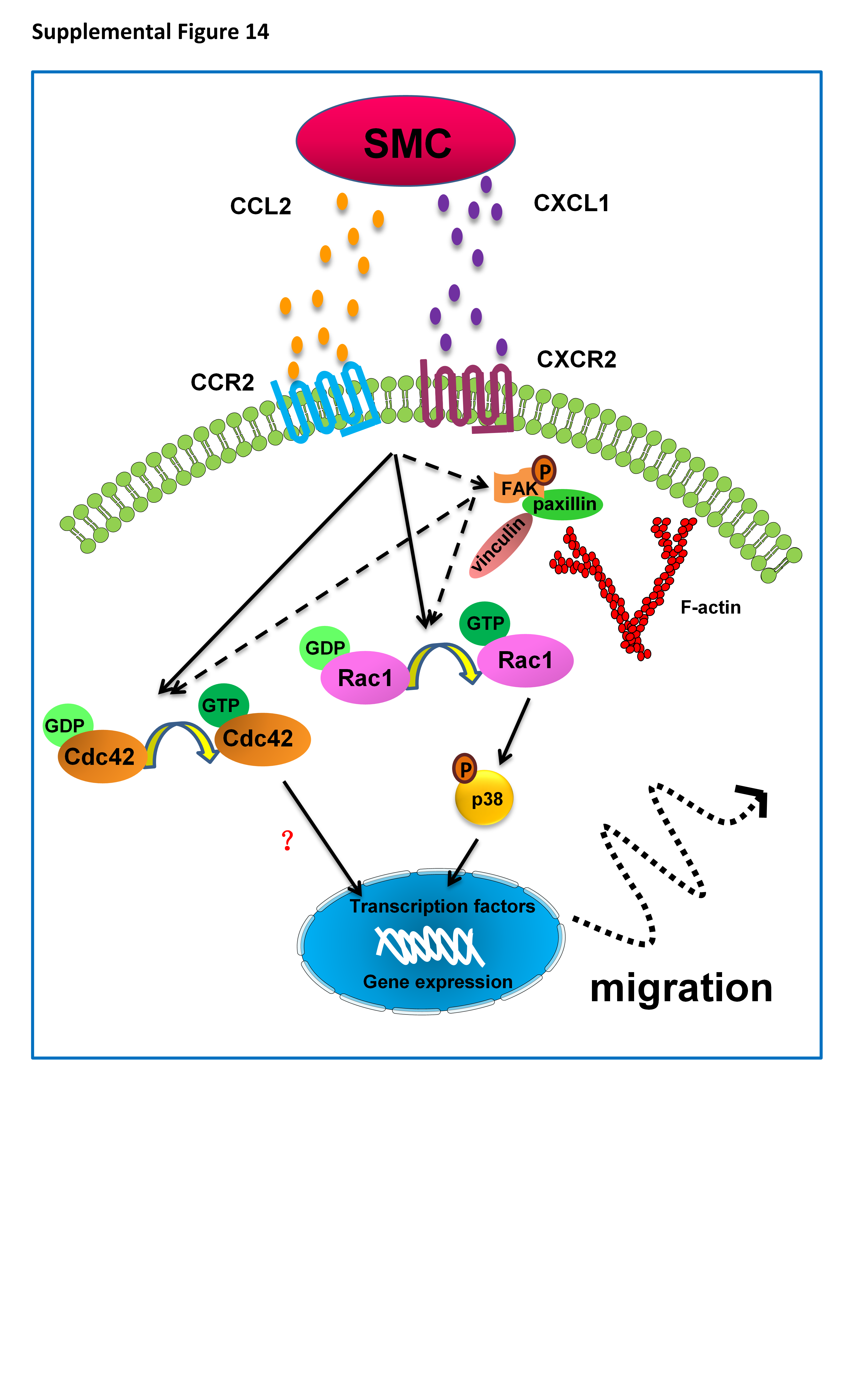


Figure 14. **Schematic illustration of the role of CCL2 and CXCL1 released from SMCs in enhancing VPCs chemotaxis.** SMCs release CCL2 and CXCL1 into the medium. When VPCs are treated with this SMC-CM, these chemokines bind to their corresponding receptors CCR2 and CXCR2 on the VPCs. The GTPase Rac1 and Cdc42 become activated and then p38 is phosphorylated via Rac1, finally leading to increased VPC migration. SMC-CM also induces expression of cytoskeleton related proteins paxillin, vinculin and phosphorylated FAK, which may also activate the Rac1 or Cdc42 signaling pathway.

Graphical Abstract. **Schematic illustration of the role of CCL2 and CXCL1 released from SMCs in enhancing VPCs chemotaxis.** SMCs release CCL2 and CXCL1 into the medium. When VPCs are treated with this SMC conditioned medium, these chemokines bind to their corresponding receptors CCR2 and CXCR2 on the VPCs. The GTPase Rac1 become activated and then p38 is phosphorylated via Rac1, finally leading to increased VPC migration. SMC conditioned medium also induces expression of cytoskeleton related proteins paxillin, vinculin and phosphorylated FAK, which may also activate the Rac1 signaling pathway.
