# Supplementary material for: Vascular Stem/Progenitor Cell Migration Induced by Smooth Muscle Cell‐Derived Chemokine (C‐C Motif) Ligand 2 and Chemokine (C‐X‐C motif) Ligand 1 Contributes to Neointima Formation
Source: Stem Cells. 2016 Jun 28;34(9):2368–80. doi: 10.1002/stem.2410 (PMC5026058; doi:10.1002/stem.2410)
Supplement: Supplementary file 4 — Supporting Information Table 1. [file STEM-34-2368-s004.docx]

**Supplemental Table 1**

| **Gene expressions of chemokines and chemotatic cytokines in different cell lines** | | | | | |
| --- | --- | --- | --- | --- | --- |
| **(2^-avgΔct^)(x10^-4^)** | | | | | |
| **Refseq** | **Symbol** | **Description** | **SMC** | **Sca-1^+^ VPC** | **clone** |
| NM_011329 | Ccl1 | Chemokine (C-C motif) ligand 1 | 7.16 | 0.83 | 0.8 |
| NM_011330 | Ccl11 | Chemokine (C-C motif) ligand 11 | 7.16 | 15.81 | 3.14 |
| NM_011331 | Ccl12 | Chemokine (C-C motif) ligand 12 | 560.56 | 0.42 | 0.46 |
| NM_011332 | Ccl17 | Chemokine (C-C motif) ligand 17 | 175.55 | 2.95 | 2.05 |
| NM_011888 | Ccl19 | Chemokine (C-C motif) ligand 19 | 7.16 | 1.6 | 1.6 |
| NM_011333 | Ccl2 | Chemokine (C-C motif) ligand 2 | 11916.83 | 7981.14 | 1677.83 |
| NM_016960 | Ccl20 | Chemokine (C-C motif) ligand 20 | 309.91 | 1.21 | 0.67 |
| NM_009137 | Ccl22 | Chemokine (C-C motif) ligand 22 | 7.16 | 0.45 | 0.58 |
| NM_019577 | Ccl24 | Chemokine (C-C motif) ligand 24 | 7.16 | 0.47 | 0.61 |
| NM_009138 | Ccl25 | Chemokine (C-C motif) ligand 25 | 100.48 | 116.09 | 73.64 |
| NM_001013412 | Ccl26 | Chemokine (C-C motif) ligand 26 | 7.16 | 0.62 | 1.15 |
| NM_020279 | Ccl28 | Chemokine (C-C motif) ligand 28 | 7.16 | 6.88 | 7.42 |
| NM_011337 | Ccl3 | Chemokine (C-C motif) ligand 3 | 600.79 | 0.42 | 0.5 |
| NM_013652 | Ccl4 | Chemokine (C-C motif) ligand 4 | 191.44 | 1.17 | 0.79 |
| NM_013653 | Ccl5 | Chemokine (C-C motif) ligand 5 | 26.1 | 263.02 | 38.74 |
| NM_009139 | Ccl6 | Chemokine (C-C motif) ligand 6 | 617.68 | 6.35 | 0.89 |
| NM_013654 | Ccl7 | Chemokine (C-C motif) ligand 7 | 25105.44 | 13329.91 | 4055.64 |
| NM_021443 | Ccl8 | Chemokine (C-C motif) ligand 8 | 34.43 | 417.52 | 7.98 |
| NM_011338 | Ccl9 | Chemokine (C-C motif) ligand 9 | 156.03 | 9.6 | 3.76 |
| NM_027022 | Cmtm2a | CKLF-like MARVEL transmembrane domain containing 2A | 7.16 | 0.42 | 0.46 |
| NM_024217 | Cmtm3 | CKLF-like MARVEL transmembrane domain containing 3 | 1464.01 | 1350.28 | 1536.8 |
| NM_153582 | Cmtm4 | CKLF-like MARVEL transmembrane domain containing 4 | 193.44 | 91.29 | 56.33 |
| NM_026066 | Cmtm5 | CKLF-like MARVEL transmembrane domain containing 5 | 7.26 | 2.62 | 0.49 |
| NM_026036 | Cmtm6 | CKLF-like MARVEL transmembrane domain containing 6 | 158.21 | 52.8 | 78.02 |
| NM_009142 | Cx3cl1 | Chemokine (C-X3-C motif) ligand 1 | 82.47 | 111.61 | 84.59 |
| NM_008176 | Cxcl1 | Chemokine (C-X-C motif) ligand 1 | 5598.06 | 4179.3 | 965.89 |
| NM_021274 | Cxcl10 | Chemokine (C-X-C motif) ligand 10 | 24.1 | 95.39 | 8.59 |
| NM_019494 | Cxcl11 | Chemokine (C-X-C motif) ligand 11 | 7.16 | 1.13 | 0.46 |
| NM_021704 | Cxcl12 | Chemokine (C-X-C motif) ligand 12 | 1222.58 | 3211.53 | 10752.46 |
| NM_018866 | Cxcl13 | Chemokine (C-X-C motif) ligand 13 | 7.16 | 0.42 | 0.46 |
| NM_019568 | Cxcl14 | Chemokine (C-X-C motif) ligand 14 | 233.25 | 8.33 | 5.32 |
| NM_011339 | Cxcl15 | Chemokine (C-X-C motif) ligand 15 | 9.82 | 44.19 | 450.6 |
| NM_023158 | Cxcl16 | Chemokine (C-X-C motif) ligand 16 | 264.24 | 249.41 | 175.14 |
| NM_009140 | Cxcl2 | Chemokine (C-X-C motif) ligand 2 | 133.04 | 3.22 | 3.83 |
| NM_203320 | Cxcl3 | Chemokine (C-X-C motif) ligand 3 | 29.56 | 0.54 | 0.75 |
| NM_009141 | Cxcl5 | Chemokine (C-X-C motif) ligand 5 | 1351.84 | 22058.48 | 34058.15 |
| NM_008599 | Cxcl9 | Chemokine (C-X-C motif) ligand 9 | 8.4 | 2.33 | 0.46 |
| NM_008337 | Ifng | Interferon gamma | 7.16 | 0.42 | 0.46 |
| NM_010551 | Il16 | Interleukin 16 | 7.57 | 95.17 | 231.64 |
| NM_008361 | Il1b | Interleukin 1 beta | 34.79 | 0.74 | 1.02 |
| NM_021283 | Il4 | Interleukin 4 | 7.16 | 0.54 | 0.63 |
| NM_031168 | Il6 | Interleukin 6 | 183 | 2128.63 | 366.85 |
| NM_019932 | Pf4 | Platelet factor 4 | 149.16 | 1.26 | 2.99 |
| NM_023785 | Ppbp | Pro-platelet basic protein | 7.16 | 2.65 | 2.72 |
| NM_011577 | Tgfb1 | Transforming growth factor, beta 1 | 386.87 | 221.69 | 210.7 |
| NM_013693 | Tnf | Tumor necrosis factor | 42.1 | 0.42 | 0.46 |
| NM_008510 | Xcl1 | Chemokine (C motif) ligand 1 | 7.16 | 0.42 | 0.46 |
