# Supplementary material for: Vascular Stem/Progenitor Cell Migration Induced by Smooth Muscle Cell‐Derived Chemokine (C‐C Motif) Ligand 2 and Chemokine (C‐X‐C motif) Ligand 1 Contributes to Neointima Formation
Source: Stem Cells. 2016 Jun 28;34(9):2368–80. doi: 10.1002/stem.2410 (PMC5026058; doi:10.1002/stem.2410)
Supplement: Supplementary file 5 — Supporting Information Table 2. [file STEM-34-2368-s005.docx]

**Supplemental Table 2**

| **Gene expressions of chemokines and chemotatic cytokines receptors in different cell lines** | | | | | |
| --- | --- | --- | --- | --- | --- |
| **(2^-avgΔct^ )(x10^-4^)** | | | | | |
| **Refseq** | **Symbol** | **Description** | **SMC** | **Sca-1^+^ VPC** | **clone** |
| NM_007577 | C5ar1 | Complement component 5a receptor 1 | 118.66 | 0.42 | 0.46 |
| NM_021609 | Ackr2 | Chemokine binding protein 2 | 7.16 | 0.65 | 0.59 |
| NM_009912 | Ccr1 | Chemokine (C-C motif) receptor 1 | 30.5 | 3.99 | 2.13 |
| NM_007721 | Ccr10 | Chemokine (C-C motif) receptor 10 | 7.16 | 3.11 | 6.23 |
| NM_007718 | Ccr1l1 | Chemokine (C-C motif) receptor 1-like 1 | 7.16 | 0.42 | 0.46 |
| NM_009915 | Ccr2 | Chemokine (C-C motif) receptor 2 | 7.29 | 0.42 | 0.51 |
| NM_009914 | Ccr3 | Chemokine (C-C motif) receptor 3 | 45.75 | 0.42 | 0.46 |
| NM_009916 | Ccr4 | Chemokine (C-C motif) receptor 4 | 7.16 | 0.42 | 0.46 |
| NM_009917 | Ccr5 | Chemokine (C-C motif) receptor 5 | 126.74 | 0.42 | 0.47 |
| NM_009835 | Ccr6 | Chemokine (C-C motif) receptor 6 | 7.16 | 0.42 | 0.48 |
| NM_007719 | Ccr7 | Chemokine (C-C motif) receptor 7 | 7.16 | 0.45 | 0.78 |
| NM_007720 | Ccr8 | Chemokine (C-C motif) receptor 8 | 7.16 | 0.42 | 0.46 |
| NM_009913 | Ccr9 | Chemokine (C-C motif) receptor 9 | 7.16 | 0.88 | 1.03 |
| NM_145700 | Ackr4 | Chemokine (C-C motif) receptor-like 1 | 7.16 | 2.02 | 3.72 |
| NM_017466 | Ccrl2 | Chemokine (C-C motif) receptor-like 2 | 7.16 | 4.99 | 10.26 |
| NM_008153 | Cmklr1 | Chemokine-like receptor 1 | 29.67 | 0.42 | 0.72 |
| NM_009987 | Cx3cr1 | Chemokine (C-X3-C) receptor 1 | 80.77 | 0.42 | 0.46 |
| NM_178241 | Cxcr1 | Chemokine (C-X-C motif) receptor 1 | 7.16 | 0.42 | 0.46 |
| NM_009909 | Cxcr2 | Chemokine (C-X-C motif) receptor 2 | 7.16 | 0.42 | 0.46 |
| NM_009910 | Cxcr3 | Chemokine (C-X-C motif) receptor 3 | 7.16 | 0.42 | 0.46 |
| NM_009911 | Cxcr4 | Chemokine (C-X-C motif) receptor 4 | 63.59 | 0.59 | 0.46 |
| NM_007551 | Cxcr5 | Chemokine (C-X-C motif) receptor 5 | 7.16 | 0.42 | 0.46 |
| NM_030712 | Cxcr6 | Chemokine (C-X-C motif) receptor 6 | 7.16 | 1.31 | 9.02 |
| NM_007722 | Ackr3 | Chemokine (C-X-C motif) receptor 7 | 145.08 | 795.5 | 779.13 |
| NM_010045 | Darc | Duffy blood group, chemokine receptor | 9.42 | 0.48 | 0.48 |
| NM_013521 | Fpr1 | Formyl peptide receptor 1 | 33.72 | 0.91 | 1.16 |
| NM_001025381 | Gpr17 | G protein-coupled receptor 17 | 7.16 | 0.87 | 1.24 |
| NM_011798 | Xcr1 | Chemokine (C motif) receptor 1 | 7.16 | 0.54 | 0.46 |
