# Supplementary material for: Vascular Stem/Progenitor Cell Migration Induced by Smooth Muscle Cell‐Derived Chemokine (C‐C Motif) Ligand 2 and Chemokine (C‐X‐C motif) Ligand 1 Contributes to Neointima Formation
Source: Stem Cells. 2016 Jun 28;34(9):2368–80. doi: 10.1002/stem.2410 (PMC5026058; doi:10.1002/stem.2410)
Supplement: Supplementary file 6 — Supporting Information Table 3. [file STEM-34-2368-s006.docx]

**Supplemental Figure 3**

| **Gene expressions of other chemotaxis in different cell lines  (2^-avgΔct^)(x10^-4^)** | | | | | |
| --- | --- | --- | --- | --- | --- |
| **Refseq** | **Symbol** | **Description** | **SMC** | **Sca-1^+^VPC** | **clone** |
| NM_010431 | Hif1a | Hypoxia inducible factor 1, alpha subunit | 1209.93 | 2109.05 | 3514.36 |
| NM_008401 | Itgam | Integrin alpha M | 89 | 0.42 | 0.46 |
| NM_008404 | Itgb2 | Integrin beta 2 | 226.87 | 15.13 | 17.62 |
| NM_011949 | Mapk1 | Mitogen-activated protein kinase 1 | 910.62 | 526.05 | 694.12 |
| NM_011951 | Mapk14 | Mitogen-activated protein kinase 14 | 159.87 | 191.66 | 208.76 |
| NM_178804 | Slit2 | Slit homolog 2 (Drosophila) | 1105.68 | 276.74 | 276.1 |
| NM_011905 | Tlr2 | Toll-like receptor 2 | 59.33 | 61.21 | 34.59 |
| NM_021297 | Tlr4 | Toll-like receptor 4 | 50.76 | 100.13 | 91.92 |
| NM_138302 | Tymp | Thymidine phosphorylase | 7.16 | 0.73 | 0.55 |
